# Competitive Photoisomerization and Energy Transfer Processes in Fluorescent Multichromophoric Systems

**DOI:** 10.1002/chem.202202071

**Published:** 2022-10-10

**Authors:** Yang Zhou, Stéphane Maisonneuve, François Maurel, Juan Xie, Rémi Métivier

**Affiliations:** ^1^ ENS Paris-Saclay Université Paris-Saclay CNRS, PPSM 91190 Gif-sur-Yvette France; ^2^ Université Paris Cité CNRS, ITODYS 75013 Paris France

**Keywords:** energy transfer, fluorescence, photoisomerization, molecular dynamics, multichromophoric systems

## Abstract

Multichromophoric systems showing both fluorescence and photoisomerization are fascinating, with complex interchromophoric interactions. The experimental and theoretical study of a series of compounds, bearing a variable number of 4‐dicyanomethylene‐2‐*tert*‐butyl‐6‐(*p*‐(*N*‐(2‐azidoethyl)‐*N*‐methyl)aminostyryl)‐4H‐pyran (DCM) units are reported. The photophysical properties of multi‐DCM derivatives, namely **2DCM** and **3DCM**, were compared to the single model azido‐functionalized **DCM**, in the *E* and *Z* isomers. The (*EE*)‐**2DCM** and (*EEE*)‐**3DCM** were synthesized via the click reaction. Steady‐state spectroscopy and photokinetics experiments under UV or visible irradiation indicated the presence of intramolecular energy transfer processes among the DCM units. Homo‐ and hetero‐energy transfer processes between adjacent chromophores were confirmed by fluorescence anisotropy and decays. Molecular dynamics simulations for **2DCM** were carried out and analyzed using a Markov state model, providing geometrical parameters (orientation and distance between chromophores) and energy transfer efficiency. This work contributes to a better understanding and rationalization of multiple energy transfer processes occuring within multichromophoric systems.

## Graphical abstract

**Multichromophoric systems** showing both fluorescence and photoisomerization were investigated by experimental and theoretical methods. We highlight the prominent role of intramolecular homo‐ and hetero‐energy transfer processes between the different isomers of the chromophores at the origin of their peculiar photophysical and photochemical properties.

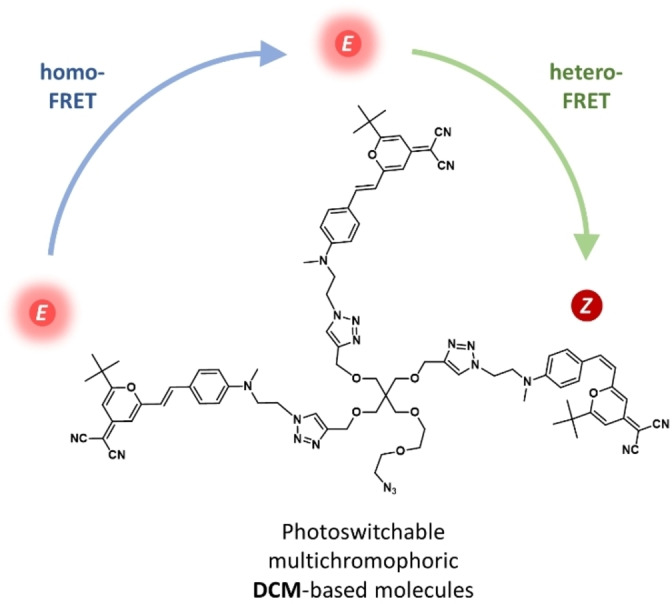

## Introduction

Multichromophic molecules and assemblies are envisioned as a promising category of functional building blocks to be applied in light‐harvesting materials, optoelectronic devices, adjustable polymers, or supramolecular systems.^[1]^ Multichromophores containing a large number of chromophores enable specific features in a wide range of fields. For example, the multichromophores including photochromic moieties (multiphotochromic compounds) and fluorophores can exhibit photoswitchable colors, high‐contrast fluorescence via resonance energy transfer (RET), and they can serve as multidimensional logic gates or induce multiple states supramolecular structural rearrangements.[^1c^, ^2^]

Besides applications involving structural changes or multi‐logic gates, several reports highlight the use of photoisomerizable units, such as azobenzenes, in multichromophoric systems to enable non‐linear optical features.^[3]^ Some studies were reported about photoisomerizable chromophore‐chromophore interactions, whereas most of them focus on the changes in the mechanical forces of films or crystalline solids, or the electronically or conjugationally influencing structures.^[4]^ As far as we know, photoisomerizable fluorophores included in large multichromophoric systems have not been explored to date.

**Figure 1:**
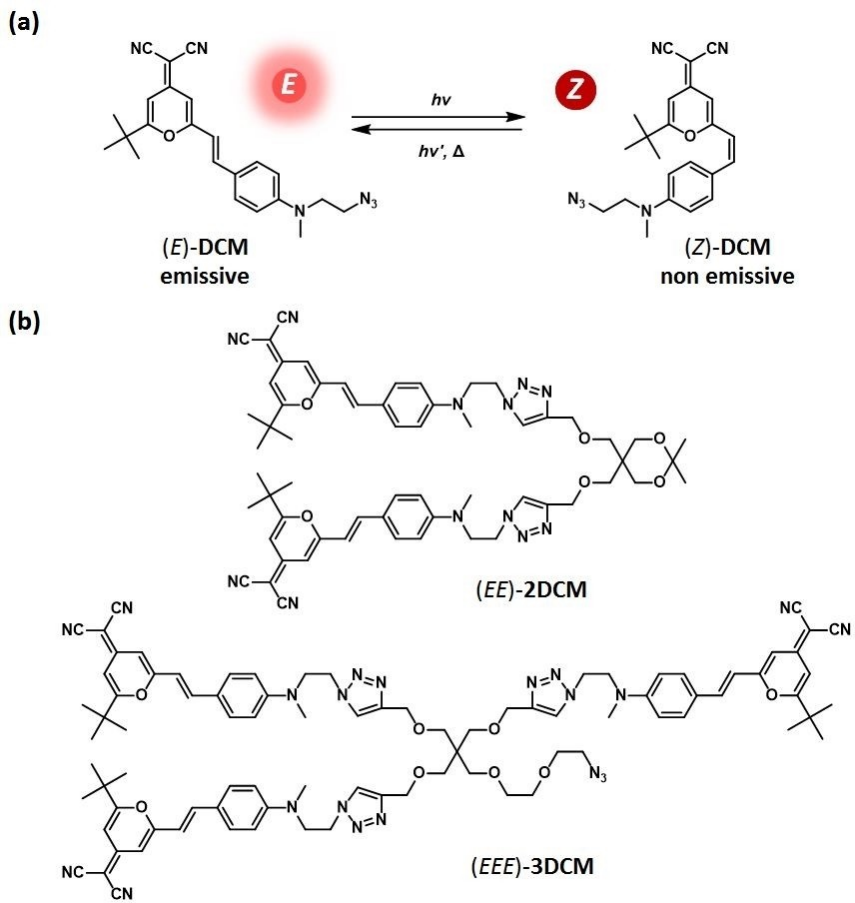
(a) *E*‐*Z* photoisomerization reaction of **DCM** and (b) structures of **2DCM** and **3DCM** derivatives with each DCM unit being in the *E* form.

4‐Dicyanomethylene‐2‐*tert*‐butyl‐6‐(*p*‐(*N*‐(2‐azidoethyl)‐*N*‐methyl)aminostyryl)‐4H‐pyran (DCM) derivatives represent one category of photoisomerizable fluorophores with a donor‐π‐acceptor character, as previously reported by our group.^[5]^ The double‐bond isomerization can be induced by light, leading to the interconversion between the emissive *E*‐form and the non‐emissive *Z*‐form. Several DCM analogues have been investigated,^[5]^ demonstrating their efficiency, fatigue resistance, and ability to serve as brightly emissive molecular photoswitches. Since the DCM molecule shows both fluorescence and photoisomerization properties, we could expect interesting interactions by Förster resonance energy transfer (FRET) processes and following, novel optical features, when merged together in larger molecules. Therefore, we designed a series of DCM‐based molecules, from the single model chromophore, **DCM**, to more sophisticated structures, **2DCM** and **3DCM**, where two and three parent azido DCMs are connected together via a central flexible dendritic linker by the cooper (I)‐catalyzed alkyne‐azide cycloaddition (CuAAC) reaction.^[6]^ The corresponding chemical structures are shown in Figure 1, where all the DCM units are represented in the *E*‐form. Such a design strategy offers the possibility to consider further functionalization with other molecular systems.

In this work, we report on the synthesis, photophysical and photochemical investigations of the **2DCM** and **3DCM** multichromophoric compounds, which are compared to the **DCM** model chromophore, by means of steady‐state absorption spectroscopy, time‐resolved fluorescence, fluorescence anisotropy, and photokinetics experiments. The photophysical properties of the series were investigated, and the comparison of their respective photokinetics under UV and visible irradiation could reveal the interchromophoric interactions existing among the DCM units. At the theoretical level, molecular dynamics (MD) simulations were performed on the **2DCM** molecule, which is a well‐representative derivative to investigate both homo‐FRET and hetero‐FRET processes, followed by Markov state model (MSM) implementation to unravel the correlation between the molecular conformational changes and their impact on the FRET efficiencies.

## Results and Discussion

### Synthetic protocols and molecular structures

**Scheme 1:**
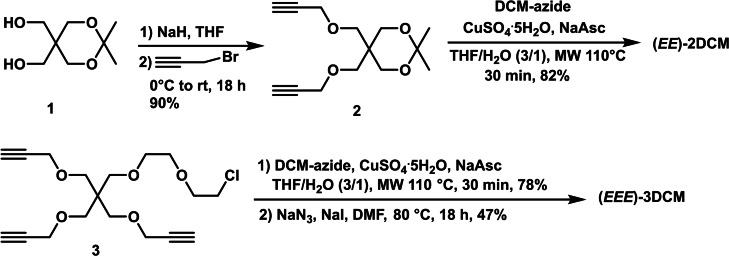
Synthesis of (*EE*)‐**2DCM** and (*EEE*)‐**3DCM**.

The design of functional dendritic architectures with well‐defined structures is a very active and exciting field of research, and many multichromophoric systems have been synthesized with dendrimers or dendrons bearing large numbers of chromophores.^[7]^ With the aim of gathering several independent DCM units, two dendritic linkers **2** and **3** with two and three propargyl groups respectively were firstly prepared (Scheme 1). Compound **2** was obtained after di‐*O*‐propargylation of isopropylidene acetal protected pentaerythritol **1**,^[8]^ while **3** was synthesized according to the reported procedure.^[9]^ CuAAC reaction of **2** with (*E*)‐**DCM** under microwave irradiation led to **2DCM** as pure (*EE*)‐isomer in 82 % yield, by shielding the ambient light during the whole synthesis procedures. For the synthesis of **3DCM**, compound **3** was firstly reacted with (*E*)‐**DCM** under microwave activation, followed by azidation with sodium azide in the presence of catalytic amount of sodium iodide in DMF at 80 °C. the (*EEE*)‐**3DCM** was obtained in 37 % total yield from **3**.

(*E*)‐**DCM** was an orange red crystal, whereas (*EE*)‐**2DCM** and (*EEE*)‐**3DCM** were obtained as red solids. The X‐ray diffraction structure of **DCM** single crystals revealed its *s‐trans‐*(*E*) configuration, as already described in our previous studies.^[5b]^ The *s‐trans*‐(*E*)‐**DCM** is the most stable form among its four identified isomers and conformers, with a planar structure. However, the crystalline ability of (*EE*)‐**2DCM** and (*EEE*)‐**3DCM** is not good enough to allow single crystal X‐ray diffraction analyses. When **2DCM** and **3DCM** are synthesized, we assume that the DCM units adopt preferentially the *s‐trans‐*(*E*) configuration, with high degrees of freedom between the DCM units allowed by the flexible central dendritic linker.

### Steady‐state spectroscopy and photokinetic studies

**Figure 2:**
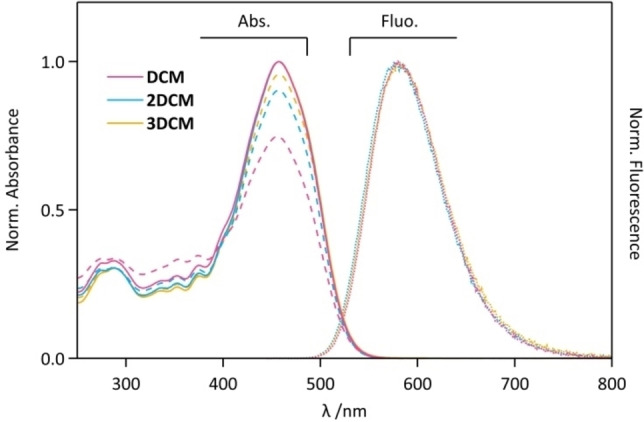
Absorption spectra after irradiation at 485 nm (dashed lines, PSS_485_) or 335 nm (full lines, PSS_335_) and emission spectra before irradiation (dotted lines) of **DCM** (pink color) **2DCM** (blue color) and **3DCM** (orange color), in THF.

According to our previous reports, THF was selected as the ideal solvent, since it allows the highest photoisomerization conversion of the DCM chromophore under irradiation, with significant emission properties.^[5a]^ The absorption and fluorescence spectra of **DCM**, **2DCM** and **3DCM** in their *E* forms in THF are depicted in Figure 2. The absorption and fluorescence show that the three compounds have similar absorption and emission bands, with their maxima located at 457 nm and 588–590 nm, respectively. Table 1 presents their molar absorption coefficient (*ϵ*) at 457 nm, with increasing values in the sequencial order **DCM**<**2DCM**<**3DCM**. However the *ϵ* increments are not exactly proportional to the number of DCM units: it is only 1.8 times higher (2.6 times, resp.) for **2DCM** (**3DCM**, resp.) than for **DCM**. The deviation from linearity of *ϵ* with the number of DCM units indicates that interchromophoric interactions between neighbouring chromophores exist in the multichromophoric systems. In addition, the fluorescence quantum yield (*Φ*_F_) was measured at 0.25 for **DCM**, but slightly decreases to 0.21 for **2DCM** and **3DCM**. Again, the decrease of *Φ*_F_ in diluted THF may be due to some level of intramolecular π‐π stacking between the DCM units within the **2DCM** and **3DCM**. It is worth noting that the DCM chromophores are only fluorescent in their *E* form, not in their *Z* form.^[5]^ After irradiation at 485 nm (inducing the *E*→*Z* forward reaction of DCM) and 335 nm (to induce the *Z*→*E* backward reaction), photostationary states (PSS) were obtained for the three compounds, showing a decrease of the main absorption band at 457 nm and a clear isosbestic point at 391 nm (Figure 2), revealing that the DCM units can be considered as simple photoswitches, either in their *E* or *Z* forms. However, major differences on the PSS photoconversion levels reached at 485 nm and 335 nm were identified through their absorption spectra. The absorbance ratio of the two PSS at 485 nm and 355 nm, A_PSS‐485_/A_PSS‐335_, measured at 457 nm, is relatively low for **DCM** (0.75) which reflects its noticeable *E*→*Z* conversion under visible irradiation. Interestingly, the **2DCM** is less prone to be isomerized to the *Z*‐form under 485 nm irradiation (A_PSS‐485_/A_PSS‐335_=0.90), whereas **3DCM** shows the lowest photoconversion (A_PSS‐485_/A_PSS‐335_=0.96). Obviously, the number of DCM units has a great impact on the *E*‐*Z* interconversion by light.

**Table 1:** Photophysical properties of **DCM**, **2DCM** and **3DCM** in THF: maximum absorption and emission wavelengths *λ*_abs_ and *λ*_em_, fluorescence quantum yields *Φ*_F_, molar absorption coefficients at 457 nm *ϵ*_(457nm)_.

	*λ*_abs_/nm	*λ*_em_/nm	*Φ*_F_^[a]^	*ϵ*_(457nm)_/L mol^−1^ cm^−1^
**DCM**	457	590	0.25±0.03	48700±600
**2DCM**	457	588	0.21±0.02	88400±1800
**3DCM**	457	590	0.21±0.02	128300±1800

[a] Emission quantum yields *Φ*_F_ were determined using Coumarine 153 in ethanol (Φ_em_=0.54)^[10]^ as the standard, *λ*_exc_=435 nm.

**Figure 3:**
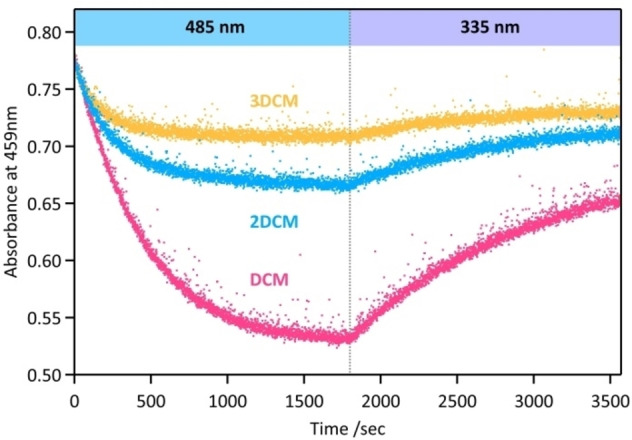
Absorbance at 459 nm of **DCM** (pink color), **2DCM** (blue color) and **3DCM** (orange color) in THF, as a function of irradiation time at 485 nm and 335 nm.

To further investigate the photoisomerization properties, we carried out photokinetic experiments under similar irradiation conditions for **DCM**, **2DCM** and **3DCM**, starting from the fresh *E*‐forms. Each compound was dissolved in THF solution, continuously probed by absorption spectra, and irradiated with visible light at 485 nm under vigorous stirring, which induced the *E*→*Z* photoisomerization of the DCM units to reach the PSS_485_. After 45 min, a subsequent irradiation at 335 nm was applied to trigger the reverse *Z*→*E* tranformation to the PSS_335_. The absorbance was monitored at 457 nm and corresponding time‐profiles are plotted in Figure 3. During the first sequence of irradiation at 485 nm, **3DCM** reaches the PSS equilibrium first (c.a. 10 min) and shows a limited decrease of absorbance (ΔAbs<0.06), whereas **DCM** presents an opposite behaviour, with as a large absorption change (ΔAbs>0.2) but a slow photokinetics, the PSS equilibrium not being reached after 30 min of irradiation. **2DCM** exhibits intermediate properties, with a PSS reached in 20 min with an absorbance decrease of ΔAbs∼0.1. During the second sequence of irradiation at 335 nm, the situation is similar, with a slow (resp. fast) photokinetics associated to large (resp. small) absorption changes for **DCM** (resp. **2DCM** and **3DCM**). This intriguing observation that the photoconversion rate and yield evolve in opposite ways, with a strong dependence on the number of DCM units in the molecules, could originate in the possible energy transfer processes that can act within chromophores in the **2DCM** and **3DCM** cases.

**Figure 4:**
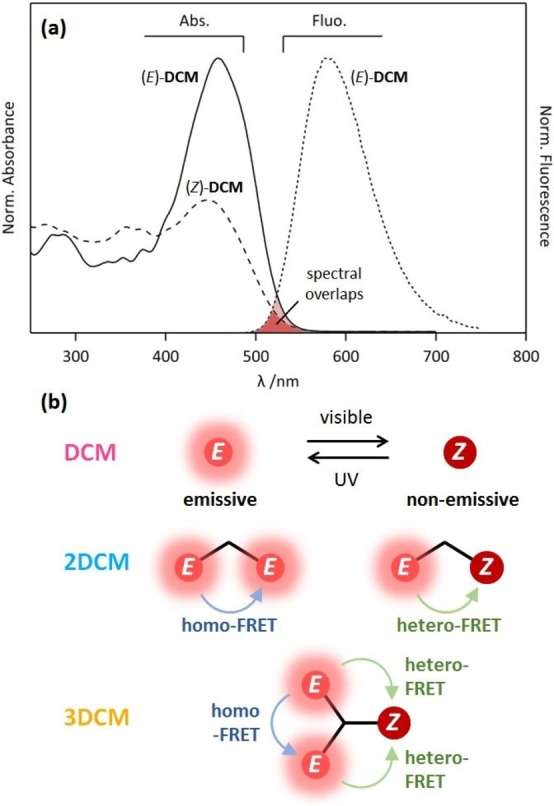
(a) Absorption spectra of (*E*)‐**DCM** and (*Z*)‐**DCM**, and emission spectrum of (*E*)‐**DCM**. Spectral overlaps are highlighted in dark and light red colors. (b) Illustration of *E* ↔ *Z* photoisomerization of **DCM** and intramolecular energy transfer processes (homo‐FRET and hetero‐FRET) occuring in **2DCM** and **3DCM**.

As a matter of fact, spectral overlaps exist between emission and absorption spectra of **DCM**, suggesting the possibility of intramolecular FRET between neighbouring chromophores in the case of **2DCM** and **3DCM** derivatives. More specifically, Figure 4 overlays the emission spectrum of (*E*)‐**DCM** with the absorption spectra of (*E*)‐**DCM** and (*Z*)‐**DCM**, revealing a small but significant spectral overlap in both cases (highlighted in red in Figure 4). Therefore, two distinct energy transfer paths can be expected in **2DCM** and **3DCM**: homo‐FRET processes between two (*E*)‐DCM chromophores, and hetero‐FRET processes between DCM units from the *E* form (donor) to the *Z* form (acceptor). As illustrated in Figure 4, when mutual orientations and interchromophoric distances are favorable, both processes can occur in the multichromophoric systems **2DCM** and **3DCM**. In the case of **3DCM**, the mixed model with two types of homo‐FRET and hetero‐FRET occurring simultaneously is also possible. Such intramolecular FRET pathways could explain the differences observed in converison yields and rates for the three DCM‐based compounds. Indeed, hetero‐FRET may represent an efficient pathway leading to the deactivation of the *E* form and promoting the formation of *Z* form excited states, which in turn would decrease the *E*→*Z* reaction efficiency and favor the *Z*→*E* reaction. Therefore, hetero‐FRET processes are expected to decrease the *E*→*Z* conversion yield under visible irradiation, but also increase the rate to reach the PSS equilibrium, through enhanced *Z*→*E* rates. This effect would be consistently higher in **3DCM**, where multiple FRET pathways are foreseen, compared to **2DCM**. In the case of **3DCM**, homo‐FRET between identical DCM units in the *E* form (hopping mechanism), can increase the probability to find an energy transfer route from the *E* form to the *Z* form (e.g. *E*→*E*→*Z* stepwise energy transfer sequence), thus indirectly promoting the excitation of the *Z* form.

### Fluorescence decays and anisotropy investigations

**Figure 5:**
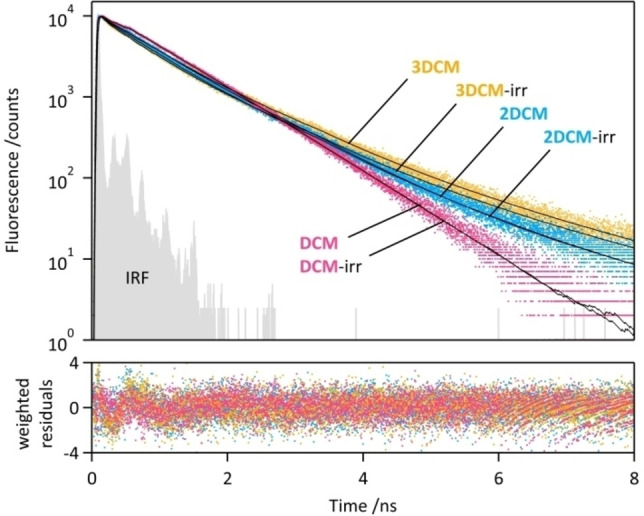
Fluorescence decay curves, instrumental response function (IRF, grey), bi‐exponential (**DCM**) and tri‐exponential (**2DCM**, **3DCM**) curve fits (black), and weighted residuals of **DCM** (pink), **2DCM** (blue) and **3DCM** (orange) in THF, before and after irradiation at 485 nm (PSS_485_). *λ*_exc_=400 nm, *λ*_em_=590 nm.

Fluorescence decay curves of the **DCM**, **2DCM** and **3DCM** were recorded by the time correlated single photon counting (TCSPC) method (Figure 5), with an excitation (resp. emission) wavelength at 400 nm (resp. 590 nm). The emission decays of the three compounds are not perfectly overlapping, with a more pronounced multiexponential character for **2DCM** and **3DCM**. The decay curves were fitted by a global analysis procedure using a bi‐exponential function for the case of **DCM**, and a tri‐exponential function for **2DCM** and **3DCM**, with satisfactory *χ* ^2^_R_<1.2 (Table 2). According to our previous studies,^[5]^ the emission arises only form the (*E*)‐DCM units. Only two components *τ*_1_=0.25 ns and *τ*_2_=0.84 ns were identified for **DCM** can be ascribed to different conformers of the *E* form, namely the *s‐trans‐*(*E*)‐**DCM** (longer decay‐time *τ*_2_) and *s‐cis‐*(*E*)‐**DCM** (short decay‐time *τ*_1_).^[5]^ An additional third component *τ*_3_=1.90 ns was found for **2DCM** and **3DCM**, associated with small contributions to the whole decays (a_3_<0.07, f_3_<0.18), which can be ascribed to a certain proportion of DCM units interacting together, through intramolecular π‐π stacking for instance, as already suggested in the previous paragraphs. Such interactions may restrict nonradiative deactivation pathways of the DCM units, resulting in a longer decay time. After irradiation at 485 nm, the decay curve of **DCM** remained unaffected, whereas **2DCM** and **3DCM** showed slightly shortened fluorescence decays: the pre‐exponential coefficient and the fraction of fluorescence intensity of the shortest decay‐time *τ*_1_ (*a*_1_, *f*_1_) increased, while those of *τ*_2_ (*a*_2_, *f*_2_) decreased, and the proportion of the third decay component *τ*_3_ remained almost stable. For **2DCM** and **3DCM**, the changes in relative weights of *τ*_1_ and *τ*_2_, corresponding to the *s‐cis* and *s‐trans* conformers of the (*E*)‐DCM units, respectively, can be tentatively explained by the conformational redistribution of the chromophores following the relaxation process after excitation. Since constraints from the linkers exist for the DCM units in **2DCM** and **3DCM**, the *s‐cis*↔*s‐trans* interconversion after the photoreaction is probably hindered by the covalent connections, which could lead to higher proportions of *s‐cis‐*(*E*) species. Unfortunately, the homo‐FRET and hetero FRET could not be confirmed by time‐resolved fluorescence because: (i) homo‐FRET is basically not expected to affect the fluorescence decays of the (*E*)‐DCM, and (ii) the (*Z*)‐DCM being non emissive, the hetero‐FRET from (*E*)‐DCM to (*Z*)‐DCM would induce a fluorescence quenching and a strong decrease of (*E*)‐DCM lifetime, down to a level below the temporal resolution of our instrumental setup (∼0.03 ns).

**Table 2:** Time‐resolved fluorescence parameters of **DCM**, **2DCM** and **3DCM** in THF, before and after irradiation at 485 nm.

	*τ*_1_ (*a*_1_, *f*_1_)	*τ*_2_ (*a*_2_, *f*_2_)	*τ*_3_ (*a*_3_, *f*_3_)	Local *χ* ^2^_R_^[a]^
**DCM**	0.25±0.08 (0.11, 0.04)	0.84±0.03 (0.89, 0.96)	–	1.02
**DCM** irr.^[b]^	0.25±0.08 (0.11, 0.03)	0.84±0.03 (0.89, 0.97)	–	1.01
**2DCM**	0.25±0.08 (0.23, 0.08)	0.84±0.03 (0.74, 0.84)	1.90±0.50 (0.03, 0.08)	1.12
**2DCM** irr.^[b]^	0.25±0.08 (0.32, 0.12)	0.84±0.03 (0.65, 0.78)	1.90±0.50 (0.04, 0.10)	1.07
**3DCM**	0.25±0.08 (0.27, 0.09)	0.84±0.03 (0.66, 0.73)	1.90±0.50 (0.07, 0.18)	1.12
**3DCM** irr.^[b]^	0.25±0.08 (0.39, 0.15)	0.84±0.03 (0.55, 0.69)	1.90±0.50 (0.06, 0.16)	1.13

[a] The global *χ* ^2^_R_ corresponding to the global analysis of the full series of decay curves was calculated at 1.08, and error bars were estimated based on a 10 % tolerance on the global *χ* ^2^_R_. [b] Irradiated samples at *λ*_irr_=485 nm.

**Figure 6:**
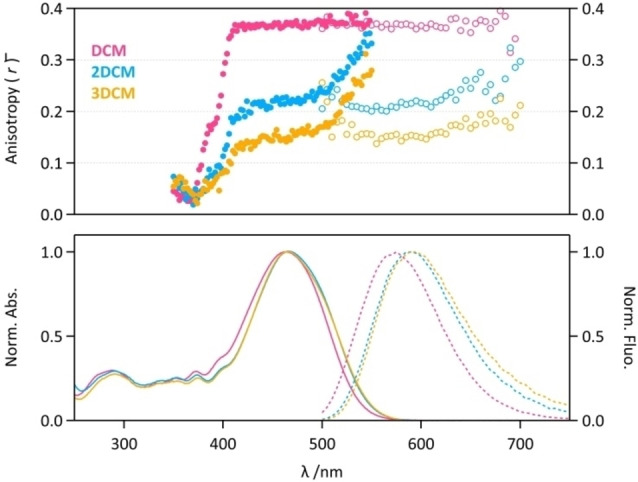
(Top) Fluorescence anisotropy of **DCM** (pink), **2DCM** (blue) and **3DCM** (orange) in propylene glycol at −45 °C, collected as a function of excitation wavelength (full circles) and emission wavelength (open circles). (Bottom) Absorption (full lines) and emission spectra (dotted lines) of **DCM** (pink), **2DCM** (blue) and **3DCM** (orange) in propylene glycol at −45 °C.

Steady‐state fluorescence anisotropy being a relevant method to investigate the homo‐FRET in multichromophoric assemblies,^[11]^ it was measured for **DCM**, **2DCM** and **3DCM** derivatives in propylene glycol at −45 °C (Figure 6), which forms a vitrified medium and freezes the molecules to avoid rotation movements during the excitation timescale. The steady‐state anisotropy ($\bar{r}$ ) of **DCM** reaches a level as high as 0.36 for *λ*_exc_>410 nm, which is very close to the maximum theoretical limit of the single‐photon excitation fluorescence anisotropy *r*_0_=0.4. This high value reflects the almost colinear absorption and emission transition dipole moment of **DCM**: the angle *β* between these transition dipole moments was calculated to be ∼15° for DCM. At *λ*_exc_ <410 nm, the anisotropy decreases down to almost zero at 360 nm, which is consistent with transition dipole moments of higher excited states. **2DCM** and **3DCM** show a steady‐state anisotropy plateau value of 0.22 and 0.18 in the range 430 nm<*λ*_exc_<500 nm (Figure 6). This depolarization compared to **DCM** clearly confirms that homo‐FRET processes do occur in **2DCM** and **3DCM**, the latter being more depolarized in the same experimental condition. At longer excitation wavelength (*λ*_exc_>500 nm), the steady‐state anisotropy rises up to 0.35 and 0.30 for **2DCM** and **3DCM**, respectively, due to the so‐called Weber's red‐edge effect: excitation in the low‐energy region of the S_0_→S_1_ electronic transition selects the DCM chromophores which are less prone to *E*→*E* homo‐FRET, and as a result, leads to an increase of the $\bar{r}$ value. Therefore, steady‐state anisotropy provides evidences of homo‐FRET within DCM units in **2DCM** and **3DCM**, and we can assume that the higher depolarization of **3DCM** emission is related to larger homo‐FRET efficiencies, due to more favorable orientation arrangements of the chromophores and/or shorter interchromophoric distances. The transition dipole moment orientations of the DCM units within multichromophoric architecture, and inter‐DCM distance parameters are critical parameters influencing the efficiency of homo‐FRET and hetero‐FRET in these systems. However, direct and dynamic measurements of such structural parameters are out of reach by conventional experimental techniques. For this reason, we decided to perform MD simulations to characterize the geometrical parameters and their influence on the FRET efficiencies.

### Modelling of the FRET parameters of 2DCM

To examine the geometrical parameters of the DCM‐DCM interaction and their effect on the homo‐FRET and hetero‐FRET efficiencies, we selected **2DCM** as the simplest molecule to be studied by MD simulations. Two models can be prepared, representing the typical FRET patterns in dendritic multichromophoric DCM‐based molecules. The first model is the (*EZ*)‐**2DCM** configuration, where we assign the (*E*)‐DCM as the donor and the other (*Z*)*‐*DCM as the acceptor in the frame of the *E*→*Z* hetero‐FRET. Besides, the (*EE*)‐**2DCM** represents a good model to investigate the *E*→*E* homo‐FRET (the donor and acceptor chromophores are chosen arbitrarily).

**Figure 7:**
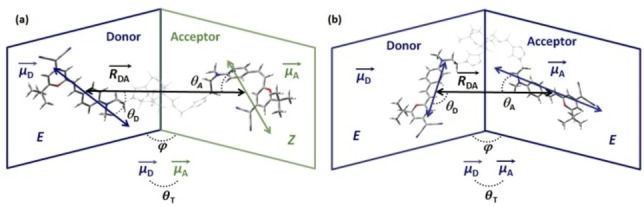
(a) (*EZ*)‐**2DCM** and (b) (*EE*)‐**2DCM**, optimized by the ONIOM method, and used as initial structures for the MD simulations. $\vec{\mu_{D}}$ refers the donor transition dipole moment vector, $\vec{\mu_{A}}$ to the acceptor transition dipole moment, $\theta_{T}$ is the angle between the two vectors, $\vec{R_{DA}}$ is the vector defined between the two mass centers of the DCM units, $\theta_{D}$ and $\theta_{A}$ are the angles between $\vec{R_{DA}}$ and $\vec{\mu_{D}}$ , $\vec{R_{DA}}$ and $\vec{\mu_{A}\;}$ , respectively, and ϕ is the dihedral angle between the ($\vec{R_{DA}}$ , $\vec{\mu_{D}}$ ) plane and the ($\vec{R_{DA}}$ , $\vec{\mu_{A}\;}$ ) plane.

Due to the large size of the studied systems, the initial structures for MD simulations were optimized using the mixed ONIOM method. The DCM units were calculated using PBE0 functional and the 6‐311G+(d,p) basis set while the bridge connecting the DCM unit was treated using the semi‐empirical PM6 method (see details in Figure S1). The optimized geometries have been built and converged in an unfolded conformation for both (*EZ*)‐**2DCM** and (*EE*)‐**2DCM** structures, as shown in Figure 7 and Figure S1. They were packed with 1500 THF molecule by Packmol (Figure S2),^[12]^ and MD simulations were performed with the GROMACS software package version 2020^[13]^ using general AMBER force field (GAFF)^[14]^ from AmberTools.^[15]^ All the simulations for (*EZ*)‐**2DCM** and (*EE*)‐**2DCM** were performed in a sequence involving the energy minimization, the equilibrium isothermal‐isobaric (constant particle number, pressure and temperature, NPT) ensemble, and the production NPT ensemble for 200 ns, which was validated by comparison with longer trajectories (Figure S5 and Table S1).

**MD simulations and FRET efficiency trajectories**. A first observation to the MD trajectories showed us that the DCM unit keep their *E* or *Z* isomeric state over the whole simulation, but could flip sometimes from *s‐cis* to *s‐trans* and vice versa (Figure S3). The instantaneous values *R*_DA_(t) of the distance between the donor‐acceptor pair and orientation factor *κ* ^2^(t) were generated by the MD software toolkit from the trajectories. In this MD section, even though energy transfer processes involve the donor species in the excited state, we assume that the ground state geometry of the multichromophoric systems can be used to estimate the FRET efficiency *E*_FRET_, by the Equation 1:

(1) $$E_{FRET}=\frac{1}{1+{\left(R_{DA}/R_{0}\right)}^{6}}$$

where *R*_0_ is the Förster radius of the donor‐acceptor pair, calculated by the Equation 2:

(2) $$R_{0}^{6}=\frac{9000\times ln\left(10\right)\times \kappa^{2}\times \Phi_{D}^{0}}{128\times \pi^{5}\times N_{A}\times n^{4}}\times J\left(\lambda \right)$$

where *n* is the refractive index of THF, *Φ*_D_^0^ is the fluorescence quantum yield of the DCM chromophore in absence of energy transfer, *J*(*λ*) refers to the absorption and emission spectral overlap integral, *N*_A_ is the Avogadro constant. The initial geometries of (*EZ*)‐**2DCM** and (*EE*)‐**2DCM** are depicted in Figure 7 and Figure S1. The *R*_DA_ was derived from the distance between atoms closest to the mass centers of each DCM fragment, and the transition dipole moment vectors of the (*E*)‐DCM and (*Z*)‐DCM fragments at their first excited states were calculated by TDDFT. The *κ* ^2^(t) was then calculated through the Equation 3:

(3) $$\kappa^{2}={\left(\sin\theta_{D}\sin\theta_{A}\cos\phi -2\cos\theta_{D}\cos\theta_{A}\right)}^{2}$$

where *θ*_D_, *θ*_A_, and *ϕ* are defined in Figure 7.

**Figure 8:**
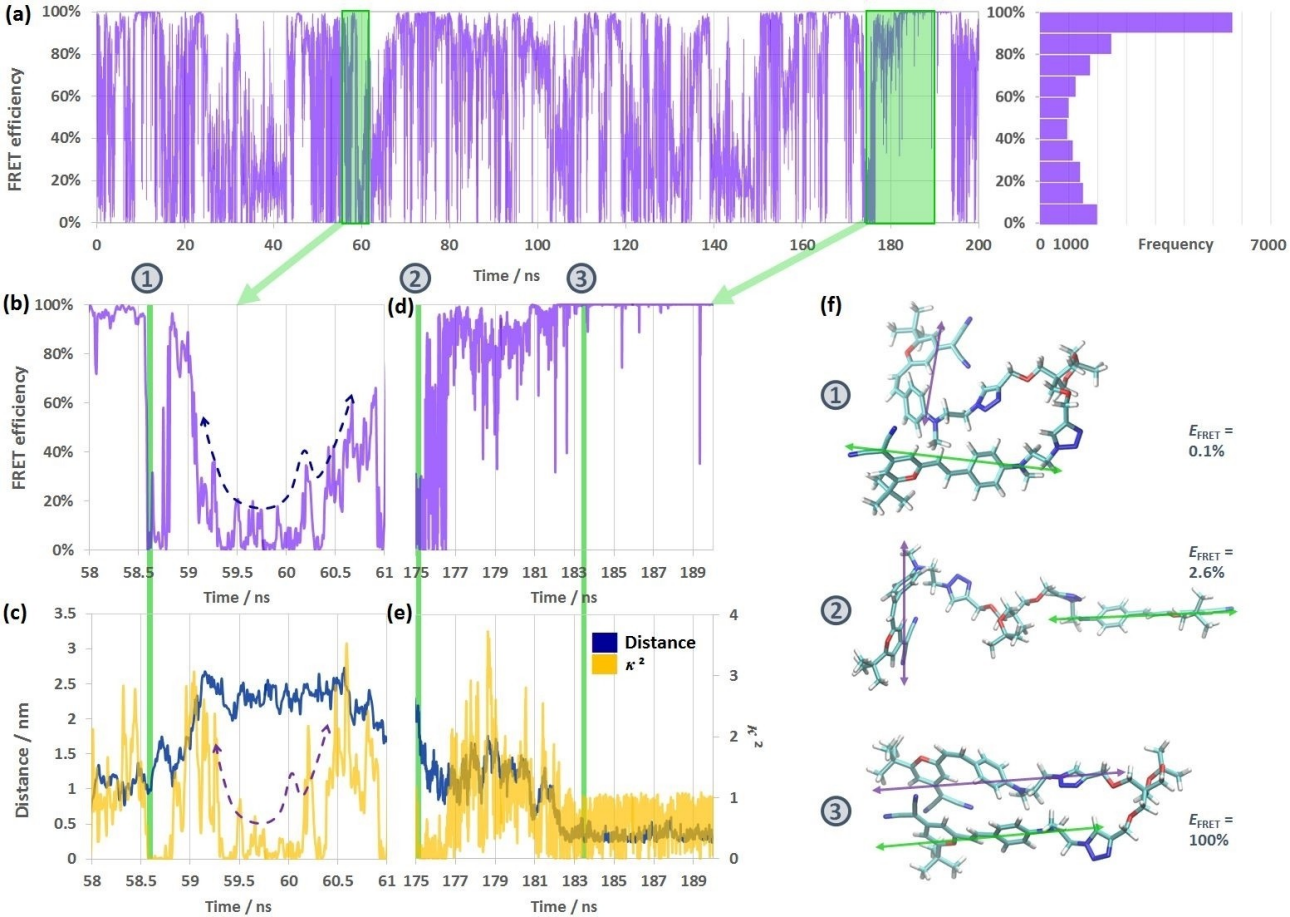
(a) MD trajectory of (*EZ*)‐**2DCM** hetero‐FRET efficiency *E*_FRET_ (t) during 200 ns, and corresponding histogram of *E*_FRET_ distribution. (b, d) Enlarged areas of the *E*_FRET_ (t) trajectory in the time intervals 58–61 ns and 175–190 ns. (c, e) Enlarged areas of the *R*_DA_(t) (navy blue) and *κ* ^2^(t) (yellow) trajectories corresponding to (b, d), respectively. (f) Three selected typical geometries of (*EZ*)‐**2DCM**, indicated by green vertical bars in (b‐e), with green and violet arrows representing the transition dipole moments of the donor (*E*)‐DCM and the acceptor (*Z*)‐DCM units, respectively. Their corresponding FRET efficiencies are marked in the insets.

The (*Z*)‐**DCM** molar absorption coefficient was determined from the DCM conversion rate $\alpha_{Z}$ , by a well‐established method in our group.^[5]^ From the **DCM** spectra shown in Figure 4a, the spectral overlap intergrals *J*(*λ*) were then calculated for (*EZ*)‐**2DCM** and (*EE*)‐**2DCM** to be 1.03×10^13^ nm^4^ M^−1^ cm^−1^ and 5.90×10^13^ nm^4^ M^−1^ cm^−1^, respectively. The instantaneous *E*_FRET_(t) can be derived from the instantaneous orientational factor *κ* ^2^(t) and the interchromophoric distance *R*_DA_(t) (corresponding histograms and average values in Figure S4). The 200 ns trajectory of *E*_FRET_(t) for (*EZ*)‐**2DCM** is shown in Figure 8a, together with the histogram of *E*_FRET_. As a result, the *E*_FRET_ vaues calculated for the (*EZ*)‐**2DCM** span from 0 % to 100 %, with an average level of 63.4 %, showing that a very wide variety of geometries are visited during the MD simulation, associated to very different FRET efficiencies. The highest probability (largest number of frames) corresponds to *E*_FRET_ in the 80–100 % range, but the statistical distribution has a very flat behaviour, with a large contribution of events where *E*_FRET_ <10 %. These extreme regions indicate the great conformational variability of the molecule, at the origin of the large dispersion of *κ* ^2^ and/or${\;R}_{DA}$ values. Figure 8b,d corresponds to enlarged areas of the *E*_FRET_ trajectory, their corresponding *R*_DA_ and *κ* ^2^ being plotted in Figure 8c,e, respectively. During some specific time‐periods (58.0–58.5 ns, 183–190 ns), the *R*_DA_ values are below 1.3 nm, pushing the FRET efficiency up to nearly 100 %, except for some extremely unfavorable orientations. For *R*_DA_<1 nm, the conformations of (*EZ*)‐**2DCM** are mostly in a folded geometry, as exemplified for the geometry #3 in Figure 8f, with *κ* ^2^ close to 1 (two transition dipole moment almost parallel to each other). When the interchromophoric distance is in the interval 1.5<*R*_DA_<2 nm, i.e. 58.5–61.0 ns in Figure 8b–c, the *κ* ^2^ starts to control the *E*_FRET_ level, the higher the *κ* ^2^, the higher the *E*_FRET_. The geometry #1 displayed in Figure 8f presents a folded structure, but an unfavored orientation of the DCM units (*κ* ^2^∼0) leading to an absence of hetero‐FRET. When the *R*_DA_ becomes higher than 1.5 nm, the *κ* ^2^ fully dominates the FRET efficiency shape: the *E*_FRET_ trajectory synchronizes the *κ* ^2^ one in Figure 8b–c, showing identical trends (two tendency dashed lines in the range 59.2‐60.7 ns, where *R*_DA_≥2).

The unfolded conformation #2 shown in Figure 8f highlights the typical situations where both large DCM‐DCM distance and almost perpendicular orientation prevent the FRET process.

In the case of (*EE*)‐**2DCM**, the MD simulation provides similar trajectories (see Figure S6), with even higher levels of homo‐FRET efficiency throughout the whole trajectory length, resulting in an average *E*_FRET_ value as high as 87.8 %. In a first approach, the larger homo‐FRET efficiency compared to hetero‐FRET efficiency is essentially due to the higher spectral overlap, *J*(*λ*) being almost six times larger (see above). From the histogram shown in the Figure S6, most frames contribute to *E*_FRET_ over 90 %. The enlarged areas of the (*EE*)‐**2DCM** trajectory highlight that the orientations and distances of the DCM fragments are rarely unfavorable to the homo‐FRET. Several examples of conformations have been selected in Figure S6, and even the unfolded form can reach an *E*_FRET_ value of 55 % since the transition dipole moments are favorably parallel.

Concerning the interchromophoric distance between the DCM units, *R*_DA_, the Table 3 lists three categories, counting the situations (frames) where *R*_DA_≤1, 1<*R*_DA_ <2 and *R*_DA_≥2. It provides a synthetic picture of conformations in the two trajectories. The *R*_DA_ ≤ 1 region of (*EZ*)‐**2DCM** reaches 91.5 % average *E*_FRET_ and reaches 98.5 % in the case of (*EE*)‐**2DCM**. This region can be viewed as mostly dominated by the interchromophoric distance, since even unparallel orientations are still favorable to FRET processes. In the intermediate 1<*R*_DA_<2 region, the average *E*_FRET_ of (*EZ*)‐**2DCM** decreases substantially (64.7 %) whereas it remains at a high value in the case of (*EE*)‐**2DCM** (92.3 %). When *R*_DA_≥2, the average *E*_FRET_ drops down in both cases (23.2 % and 61.5 % respectively), and the *κ* ^2^ starts to control the *E*_FRET_ level, as described previously.

**Table 3:** Average energy transfer efficiency value (*E*_FRET_) classified as a function of the interchromophoric distance (*R*_DA_), extracted from (*EZ*)‐**2DCM** and (*EE*)‐**2DCM** MD simulation trajectories.

	average *E*_FRET_		
	*R*_DA_≤1	1<*R*_DA_<2	*R*_DA_≥2
(*EZ*)‐**2DCM**	91.5 %	64.7 %	23.2 %
(*EE*)‐**2DCM**	98.5 %	92.3 %	61.5 %

Therefore, the MD simulations confirm that hetero‐FRET and homo‐FRET processes can take place within chromophores of (*EZ*)‐**2DCM** and (*EE*)‐**2DCM**, and the trajectory‐based instantaneous FRET analysis reveals that the completely folded conformation is the most favorable to FRET, which can reach almost 100 %. Based on the above statements, we further continued statistical implementations on the MD simulation data to identify the most representative geometries using the principal component analysis (PCA) method (Figures S7–8). More interestingly, connections between the main conformations found by PCA of (*EZ*)‐**2DCM** and (*EE*)‐**2DCM**, can be revealed by the Markov State Model (MSM) method, as detailed below.

**Figure 9:**
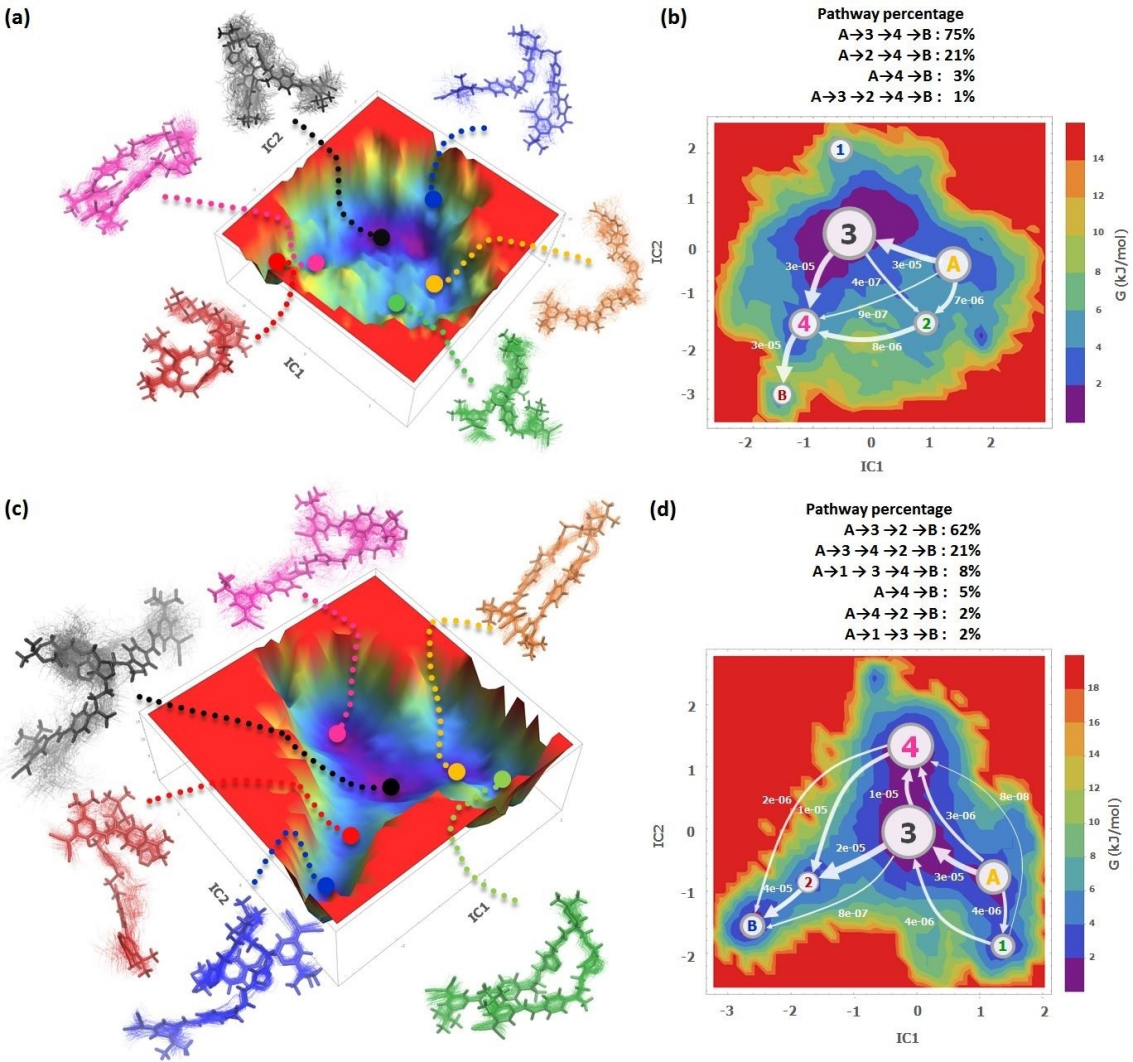
(a) tICA based on 200 ns MD trajectories of (*EZ*)‐**2DCM** and (b) its tICA‐FEL visualized MSM. (c) tICA based on 200 ns MD trajectories of (*EE*)‐**2DCM** and (d) its tICA‐FEL visualized MSM. The *κ*‐means clustering method generated 200 microstates that are lumped into 6 metastable states by the PCCA+ algorithm. The metastable states trajectories are clustered to extract the major conformations in six colors, which correspond to the labels in the transition pathway maps. The size of the labels and the arrows in (b, d) correspond to their proportional coarse‐grained fluxes. The percentages of transition pathways are listed above each MSM in (b) and (d).

**Markov State Models (MSMs) of 2DCM**. To further rationalize the conformational changes occuring during the trajectories produced by MD simulations, we built the MSM for both trajectories, corresponding to (*EZ*)‐**2DCM** and (*EE*)‐**2DCM**. The MSM reveals the important intermediate states from the MD simulation data and their mutual relationships. The analysis was carried out using the PyEMMA package.^[16]^ The MD trajectories were taken into a time‐lagged independent component analysis (tICA), and featurized by the software. The tICA maximizes the autocorrelation of the linearly transformed coordinates by a lag time *τ*, finding a maximally slow reaction in the MD simulation corresponding to the lag time of the input parameter as the first independent component (IC). This method allows to identify all conformational components even with insufficient sampling. A lag time of 1 ns was selected for each trajectory (for time lags longer than 1 ns, the timescale curves level off, for both (*EZ*)‐**2DCM** and (*EE*)‐**2DCM**, as shown in Figure S9). Furthermore, the Chapman‐Kolmogorov tests for the two MSMs show that the lag time *τ*=1 ns model accurately predicts the behaviors at longer timescales. The resulting 2D and 3D free energy landscapes (FELs) of (*EZ*)‐**2DCM** and (*EE*)‐**2DCM** configurations along their first and second independent components are shown in Figure 9. The principal component analysis (PCA) was carried out, as shown in the Figures S7–8. The 200 ns trajectories for both 2DCMs have rather low cosine contents of the first two principal components (PCs): (*EZ*)‐PC1=0.03, (*EZ*)‐PC2=0.06, (*EE*)‐PC1=0.16, (*EE*)‐PC2<0.01, which indicates an acceptable sampling of this duration.

The MSMs were obtained with 200 microstates using *κ*‐means clustering method and further lumped into 6 metastable states using the Perron cluster analysis (PCCA+) method, respectively. These metastable states were clustered to extract the major conformations of (*EZ*)‐**2DCM** and (*EE*)‐**2DCM**, as depicted in Figure 9a,c with several colors. The locations of the representative conformations from the metastable states are marked on the 3D FELs. The MSMs with transition pathways and pathway percentages on 2D FELs are displayed in Figure 9b,d.

In the (*EZ*)‐**2DCM** MSM (Figure 9a‐b), the main transition pathway starts from the unfolded form *EZ*‐A, transits through the completely folded *EZ*‐3 and *EZ*‐4, and finally ends with the more compact rolled *EZ*‐B, with a total pathway percentage of 75 %. Another pathway, counting for 21 %, omits the *EZ*‐3, getting twisted conformations directly (*EZ*‐2) and then converges into *EZ*‐4 and *EZ*‐B. We can clearly observe that this is a process which continuously shrinks the interchromophoric distance. The *EZ*‐A unfolded types generally correspond to *R*_DA_>2, whereas the most compact *EZ*‐1 and *EZ*‐B have *R*_DA_<1, as visualized in the last section, and the intermediates have geometries with 1<*R*_DA_<2 frames. Consequently, the average FRET efficiency increases along the pathways. The other minor routes with lower fluxes follow the same pattern as well, with decreasing *R*_DA_ from the unfolded conformations, thus improving the *E*_FRET_ along the path.

On the (*EE*)‐**2DCM** side (Figure 9c–d), the situation is different. The unfolded forms *EE*‐3 are the most probable intermediates during the process, and the MSM indicates a “folded conformations”→“unfolded conformations”→“single folded conformations” chain. The single folded conformations (*EE*‐2 or *EE*‐4) converge into the *EE‐*B, for about 83 % of the pathway flux. The pathways are based on the shift of the single folded and folded conformations via the unfolded conformations. Besides, other paths omitting the unfolded *EE*‐3, directly through *EE*‐2 and *EE*‐4, can occur as well. In addition, 8 % pathway flux is induced by the rolled folded types from the starting *EE*‐A.

We explored the MD simulation trajectories with both MSM (tICA) methods (and PCA, see Figures S7–8), extracting the pathways along the typical conformations of **2DCM**. The representative conformations in the FELs can be categorized according to their *R*_DA_, which are in turn related to the FRET efficiencies. In the case of (*EZ*)‐**2DCM**, the evolution process from the unfolded forms to the completely/rolled folded conformations results in nearly 70 % enhancement of the FRET efficiency. (*EE*)‐**2DCM** undergoes an increase of FRET efficiency by 30 % as long as its *R*_DA_ starts to shrink. Connected to the fluxes and pathway percentages of MSMs, the conformational fluctuation causing FRET efficiency variations in THF solution can be deduced.

## Conclusion

We have synthesized and characterized a series of DCM‐based molecules, namely **DCM**, **2DCM** and **3DCM**, to serve as first specimens and models of photoisomerizable multichromophoric assembies, and to investigate their behaviors under irradiation. Although possessing very similar absorption and emission spectral features, lower photoconversion yields and faster photokinetics were observed for **2DCM** and **3DCM** compared to **DCM**, which originate from intramolecular energy transfer processes. The homo‐FRET was demonstrated to occur. By means of steady‐state fluorescence anisotropy, an increasing depolarization of the emission was observed for **2DCM** and **3DCM**, demonstating the occurence of homo‐FRET within the systems. Although time‐resolved fluorescence cannot determine precisely the hetero‐FRET, we selected the **2DCM** molecule as a model to carry out MD simulations in two different states, (*EZ*)‐**2DCM** and (*EE*)‐**2DCM**. Thanks to the MD simulations, we could rationalize the connections between the FRET efficiency, the interchromophoric distances, orientation factors, explaining the reasons of the FRET fluctuations in time. Statistically, higher efficiencies FRET were identified for homo‐FRET (87.8 % average) than for hetero‐FRET (63.4 % average). The MSMs methodology and the statistics based on FELs, related to the MD simulations data, provided the main transition pathways among the structural conformations. In summary, we elucidated the strong FRET dependence on the molecular conformational changes. We can conclude that the photoisomerizable multichromophoric molecular architectures based on DCM chromophores are prone to be strongly affected by intramolecular energy transfer processes (homo‐ and hetero‐FRET), with tuneable properties under irradiations. The future designs on multi‐DCM systems should take into account the structural and geometrical factors (distances, orientations) to achieve desired performances.

## Experimental Section

**Materials**: The synthetic protocols of **DCM**, **2DCM** and **3DCM** are given in the Supporting Information. All commercially available compounds and solvents are purchased from Sigma‐Aldrich affiliated to Merck KGaA, Carlo Erba Reagents, TCI chemicals and Acros Organics affiliated to Thermo Fisher Scientific without further purifications. ^1^H, ^13^C, COSY, HSQC NMR data were acquired by JEOL ECS‐400 spectrometer with a broadband probe. Spectroscopic measurements were performed with spectroscopic grade solvents from Carlo Erba Reagents and Sigma‐Aldrich affiliated to Merck KGaA.

**Steady‐state spectroscopy**: Absorption spectra at room temperature were recorded using Agilent Cary‐4000 or Cary‐5000 UV spectrometers. Fluorescence emission and anisotropy data were measured with Horiba Jobin‐Yvon Fluorolog‐3 spectrofluorometer equipped with polarizers in excitation and emission. Quartz cuvettes with a 1 cm path length were used. Fluorescence emission quantum yields were determined using Courmarine 153 in ethanol (Φem=0.544) as the reference. Steady‐state anisotropy was recorded by means of the *I_VV_, I_VH_, I_HV_ and I_HH_* measurements (*V*, vertical; *H*, horizontal) of the sample at −45 °C in propylene glycol, after calibration of the polarizers with a Ludox scattering suspension.

**Photoisomerization studies**: Irradiation experiments were performed at room temperature, with a Hamamatsu Hg−Xe lamp LC8 (Lightningcure 200 W) excitation source. The desired wavelengths were selected using appropriate Semrock interferential filters at 335 nm and 485 nm. The incident lamp power was measured by means of an Ophir PD300‐UV photodiode, subtracting the residual NIR contribution from the total value. Photoisomerization kinetics were determined using a home‐made setup allowing the collection of absorption spectra at high rates under continuous irradiation. A Xenon lamp (75 W) was used as a probe light source placed at 90° with respect to the irradiation beam. Spectra were recorded every 0.2 seconds, with a spectrometer coupled with a CCD (Roper Scientific and Princeton Instruments, respectively).

**Time‐resolved fluorescence spectroscopy**: Fluorescence decay curves were obtained by the time‐correlated single‐photon counting (TCSPC) method. The setup is composed of a titanium sapphire Ti : Sa oscillator (Spectra Physics, Maï Taï) emitting pulses of 100 fs duration for the fundamental laser beam, at 80 MHz frequency. The repetition rate is reduced down to 4 MHz by a pulse picker which implements an acousto‐optic modulator. Non‐linear SHG crystals generate the desired wavelength (GWU Lasertechnik, UHG‐23‐PSK), then the beam is directed to the sample solution after adjusting the excitation power with an intensity attenuator filter wheel. Fluorescence photons are detected at 90° through a polarizer at the magic angle and a monochromator, by means of a micro‐channels plate photomultiplier (MCP‐PMT R3809 U‐50, Hamamatsu), connected to a TCSPC module (SPC‐630, Becker & Hickl). Fluorescence decays were finally processed with the help of the global non‐linear least‐squares minimization method including reconvolution analysis (Globals, Laboratory for Fluorescence Dynamics at the University of California, Irvine).

**Quantum chemical geometry optimizations**: The **DCM** and DCM units in the **2DCM** and **3DCM** were optimized by density functional theory (DFT) by using Gaussian 16 software package.^[17]^ The **2DCM**s were optimized by the ONIOM strategy in Gaussian 16, partitioning DCM units into DFT and the dendritic linker part into PM6 semi‐empirical functional. PBE0 was used for all **DCM** and DCM units at 6‐311G+(d,p) basis set level. IEFPCM solvent model of THF is used. The optimized geometries were taken into the MD simulation input set‐ups.

**Molecular Dynamics (MD) simulations**: The MD simulation inputs were based on the geometries optimized in quantum chemistry level. The electrostatic potentials of 2DCMs were calculated at B3LYP/def2‐SVP level of theory by following the restrained electrostatic potential (RESP) procedure. Each configuration of **2DCM** was encapsulated with 1500 THF molecules by Packmol program,^[12]^ setting the mutual distance tolerance limit at 2 Å, and modelled by AmberTools^[15]^ with general AMBER force field (GAFF).^[14]^ The MD simulation was carried out using GROMACS software package version 2020. 2^[13]^ trajectories of (*EE*) and (*EZ*) configured **2DCM** were obtained. All trajectories were written every 5 ps for analysis. The production ensembles use the leapfrog integrator with the step size of 2 fs, a reference temperature at 298 K, a reference pressure at 1 bar.

**Molecular Dynamics (MD) analyses**: The **2DCM** trajectories were pretreated by GROMACS toolkit (principal components analysis, PCA). The covariance matrix and following eigenvalues, eigenvectors were generated and calculated by GROMACS toolkit or PyEMMA. The all‐trajectory clustering was realized in VMD.^[18]^ The MSM was obtained from the MD simulation data by analyzing with program PyEMMA.^[16]^ The MD simulation data of **2DCM** coordinates was estimated by computing a time‐lagged independent component analysis (tICA). The Markov State Model (MSM) was validated by the methods provided from PyEMMA, including the implied timescale test and Chapman‐Kolmogorov test (Figure S10). The free energy landscapes (FELs) were drawn in Wolfram Mathematica. After getting the probability density P(x), we apply the equation below to calculate the Gibbs free energy with Boltzmann constant *k*_B_.

## Conflict of interest

The authors declare no conflict of interest.

1

## Supporting information

As a service to our authors and readers, this journal provides supporting information supplied by the authors. Such materials are peer reviewed and may be re‐organized for online delivery, but are not copy‐edited or typeset. Technical support issues arising from supporting information (other than missing files) should be addressed to the authors.

Supporting Information

## Data Availability

The data that support the findings of this study are available from the corresponding author upon reasonable request.
