# Supplementary material for: Competitive Photoisomerization and Energy Transfer Processes in Fluorescent Multichromophoric Systems
Source: Chemistry. 2022 Oct 10;28(68):e202202071. doi: 10.1002/chem.202202071 (PMC10092411; doi:10.1002/chem.202202071)
Supplement: Supplementary file 1 — Supporting Information [file CHEM-28-0-s001.pdf]

Chemistry–A European Journal

Supporting Information

Competitive Photoisomerization and Energy Transfer Processes in Fluorescent Multichromophoric Systems

Yang Zhou, Stéphane Maisonneuve, François Maurel,* Juan Xie,* and Rémi Métivier*

1. GENERAL EXPERIMENTAL **2**

2. SYNTHETIC PROCEDURES **3**

SYNTHESIS OF COMPOUND 2 3
SYNTHESIS OF COMPOUND (*EE*) 2DCM 3
SYNTHESIS OF COMPOUND 4 4
SYNTHESIS OF COMPOUND (*EEE*) 3DCM 5

3. MOLECULAR DYNAMICS (MD) SIMULATIONS **6**

OPTIMIZED GEOMETRIES OF (*EZ*)-2DCM AND (*EE*)-2DCM 6
SOLVENT CUBIC BOX WITH (*EZ*)-2DCM 7
MD DATA ANALYSIS FOR (*EZ*)-2DCM AND (*EE*)-2DCM 8
MD TRAJECTORIES AND RELATED DATA ANALYSES 10
FREE-ENERGY LANDSCAPE (FEL) BASED ON PRINCIPAL COMPONENTS ANALYSIS (PCA) 12
IMPLIED TIMESCALES FOR THE MARKOV MODEL OF (*EZ*)-2DCM AND (B) (*EE*)-2DCM 14
CHAPMAN-KOLMOGOROV TEST CURVES OF (*EZ*)-2DCM AND (B) (*EE*)-2DCM 14

4. NMR SPECTRA **15**

NMR SPECTRA OF COMPOUND 2 IN $CDCl_3$ 15
NMR SPECTRA OF COMPOUND (*EE*)-2DCM IN $CDCl_3$ 16
NMR SPECTRA OF COMPOUND 4 IN $CDCl_3$ 17
NMR SPECTRA OF COMPOUND (*EEE*)-3DCM IN $CDCl_3$ 18

1. General experimental

Commercially available solvents and reagents were used without further purification. The reactions carried out under anhydrous conditions were performed under argon. DMF and THF were previously dried through alumina or molecular sieves cartridge using a solvent purification system from MBRAUN SPS-800. Reactions were monitored by TLC on Silica Gel 60F-254 plates with detection by UV (254 nm or 365 nm) or by spraying with 10% H_2SO_4 in EtOH and heating about 30 s at 400-600 °C.

Column chromatography purifications were performed on silica gel or with a CombiFlash® Rf+ purification system using RediSep® RF or RF Gold normal phase silica columns with UV-Vis detection.

Microwave reactions were realized in a reactor of 30 mL sealed with a septum using a Monowave 300 microwave synthesis reactor from Anton Paar GmbH.

^{1}H and ^{13}C-NMR spectra were recorded on a JEOL ECS-400 spectrometer (399.78 MHz for ^{1}H, and 100.53 MHz for ^{13}C) equipped with an auto-tunable broad band probe. Chemical shifts (δ) are reported in part per million (ppm) relative to the residual solvent pic or $Si(CH_3)_4$.[1,2] Coupling constants are reported in Hertz (Hz), and the attribution of the signals is given with the following abbreviations: s (singlet), d (doublet), t (triplet), q (quartet), m (multiplet), br (broad).

HRMS (ESI) spectrum was recorded on a Q-TOF mass spectrometer by the "Fédération de Recherche" ICOA/CBM (FR2708) platform. The adducts are described as in the specific literature.[3]

[1] H. E. Gottlieb, V. Kotlyar, A. Nudelman ; *J. Org. Chem.* **1997**, 62, 7512-7515.
[2] G. R. Fulmer, A. J. M. Miller, N. H. Sherden, H. E. Gottlieb, A. Nudelman, B. M. Stoltz, J. E. Bercaw, K. I. Goldberg ; *Organometallics* **2010**, 29, 2176-2179.
[3] H. Tong, D. Bell, K. Tabei, M.M. Siegel ; *J. Am. Soc. Mass Spectrom.* **1999**, 10, 1174-1187.

2. Synthetic procedures

Synthesis of compound 2

2,2-Dimethyl-5,5-bis((prop-2-yn-1-yloxy)methyl)-1,3-dioxane (2)

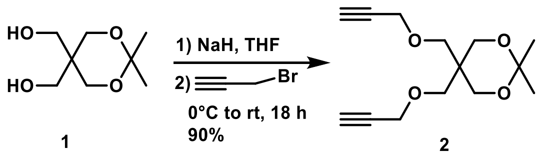

To a solution of compound **1**[4,5] (114 mg, 0.65 mmol) in THF (10 mL), sodium hydride (60% dispersion in mineral oil, 2.4 equiv.) was added in portions under argon at 0°C. After addition, the reaction mixture was stirred for 30 min, propargyl bromide (80% in toluene, 6 equiv.) was added dropwise to the reaction mixture which was then stirred at room temperature for 18 h. Saturated NH_4Cl aq. was added to quench the reaction and the reaction mixture was extracted by ethyl acetate (20 mL) for 3 times. The organic layer layers were combined and washed with brine, dried over anhydrous $MgSO_4$, filtered and concentrated. The crude product was purified by CombiFlash® chromatography with an eluent of petroleum ether and ethyl acetate (8:1) to afford **2** as a transparent liquid (147 mg, 90%). ^{1}H NMR (400 MHz, $CDCl_3$): δ (ppm) = 4.14 (d, *J* = 2.3 Hz, 4H, C*H₂*), 3.75 (s, 4H, C*H₂*), 3.53 (s, 4H, C*H₂*), 2.43 (t, *J* = 2.5 Hz, 2H, C≡C*H*), 1.41 (s, 6H, C*H₃*). ^{13}C NMR (100 MHz, $CDCl_3$): δ (ppm) = 98.27, 79.85 (C_q); 74.46 (C≡*C*H), 69.29, 62.66, 58.77, 38.87 (CH_2); 23.83 (CH_3). *m/z* $[M+Na]^+$ calcd. for $[C_{14}H_{20}NaO_4]^+$: 275.1254; found 275.1254.

Synthesis of compound (*EE*) 2DCM

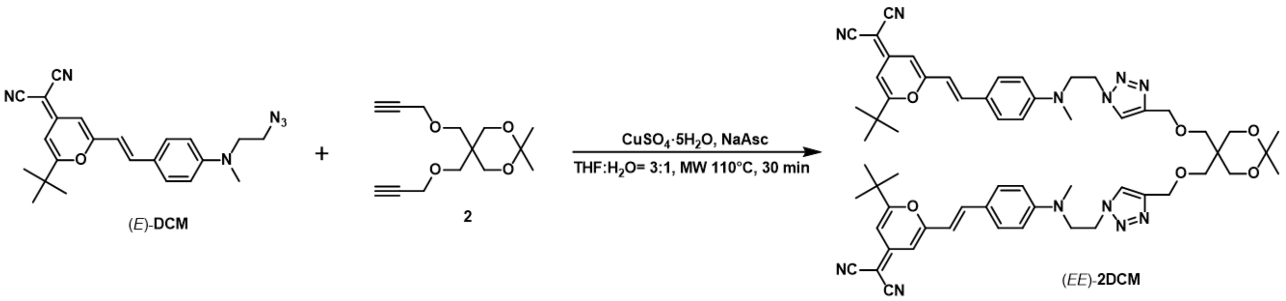

Compound **2** (33 mg, 0.13 mmol), (*E*)-**DCM**[6] (2.2 equiv.), $CuSO_4{\cdot}5H_2O$ (0.4 equiv.) and sodium ascorbate (0.8 equiv.) were added together in a mixed solvent THF/H_2O (4 mL, 3:1, v/v) to a microwave reactor tube (glass, 30 mL). The reaction mixture was kept irradiated with microwave, holding at 110°C for 30 minutes. After the reaction mixture returned to room temperature, it was poured into 0.1 M EDTA aqueous solution. The mixture was extracted with ethyl acetate (30 mL) for 3 times. The organic layers were combined and washed with brine, dried over anhydrous $MgSO_4$ and filtered. After evaporation of the solvent, the crude product was purified by

[4] C. M. Murguía, S. E. Vaillard, R. J. Grau, *Synthesis* **2001**, *7*, 1093-1097.
[5] V. Percec, P. Leowanawat, H.-J. Sun, O. Kulikov, C. D. Nusbaum, T. M. Tran, A. Bertin, D. A. Wilson, M. Peterca, S. Zhang, N. P. Kamat, K. Vargo, D. Moock, E. D. Johnston, D. A. Hammer, D. J. Pochan, Y. Chen, Y. M. Chabre, T. C. Shiao, M. Bergeron-Brlek, S. André, R. Roy, H.-J. Gabius, P. A. Heiney, *J. Am. Chem. Soc.* **2013**, *135*, 9055-9077.
[6] K. Ouhenia-Ouadahi, R. Métivier, S. Maisonneuve, A. Jacquart, J. Xie, A. Léaustic, P. Yu, K. Nakatani, *Photochem. Photobiol. Sci.*, **2012**, 11, 1705-1714.

CombiFlash® chromatography with dichloromethane and ethanol as eluent (gradient, 100:0 to 90:10, v/v) to afford (*EE*)-**2DCM** as red powder (113 mg, 82%).

^{1}H NMR (400 MHz, $CDCl_3$): δ (ppm) = 7.50 (s, 2H, triazole-*H*), 7.43-7.41 (m, 4H), 7.33 (s, 1H), 7.29 (s, 1H), 6.63 (d, *J* = 8.8 Hz, 4H, CH_{Ar}), 6.62-6.60 (m, 2H, C*H*=), 6.53-6.49 (m, 4H, C*H*=), 4.60 (t, *J* = 6.2 Hz, 4H, CH_2), 4.56 (s, 4H, CH_2), 3.94 (t, *J* = 6.0 Hz, 4H, CH_2), 3.66 (s, 4H, CH_2), 3.43 (s, 4H, CH_2), 2.88 (s, 6H, NCH_3), 1.37 (s, 18H, *t*-Bu), 1.36 (s, 6H, CH_3). ^{13}C NMR (100 MHz, $CDCl_3$): δ (ppm) = 172.08, 160.02, 156.91, 149.89, 145.52 (C_q); 137.98, 129.97, 123.55 (CH_{Ar}); 115.82, 115.71 (C_q); 114.02 (CH_{Ar}); 112.10, 105.95, 102.58 (CH=); 98.37 (C_q); 69.65, 64.96, 62.91 (CH_2); 58.32 (C_q); 52.56, 47.69 (CH_2); 38.89 (CH_3), 36.83 (C_q, *t*-Bu); 28.28, 23.88 (CH_3). HRMS (ESI-HRMS): *m/z* $[M+H]^+$ calcd. for $[C_{60}H_{69}N_{12}O_6]^+$: 1053.5458; found 1053.5454.

Synthesis of compound 4

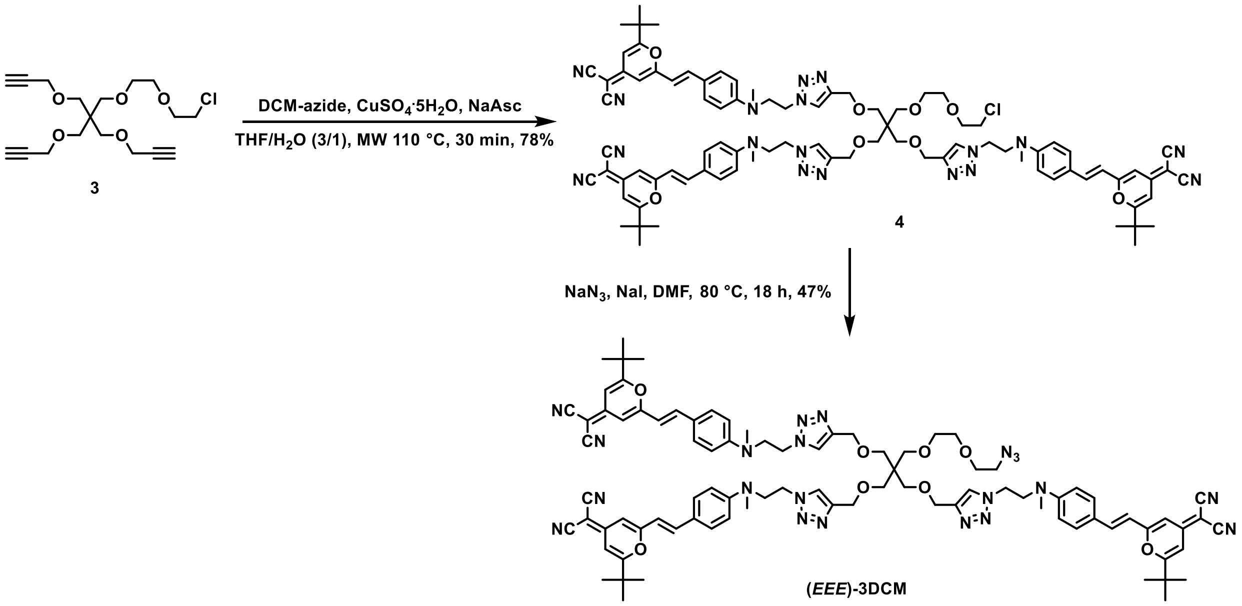

Compound **3**[7] (50 mg, 0.14 mmol), (*E*)-**DCM** (3.3 equiv.), $CuSO_4{\cdot}5H_2O$ (0.4 equiv.) and sodium ascorbate (0.8 equiv.) were added together in a mixed solvent THF/H_2O (4 mL, 3:1, v/v) to a microwave reactor tube. The reaction mixture was kept irradiated with microwave, holding at 110°C for 30 minutes. After the reaction mixture returned to room temperature, it was poured into 0.1 M EDTA solution. The mixture was extracted with ethyl acetate (30 mL) for 3 times. The organic layers were combined and washed with brine, dried over anhydrous $MgSO_4$ and filtered,. After evaporation of the solvent, the crude product was purified by CombiFlash® chromatography with dichloromethane and ethanol as eluent (gradient, 100:0 to 90:10, v/v) to afford compound **4** as a red powder (170 mg, 78%).

^{1}H NMR (400 MHz, $CDCl_3$): δ (ppm) = 7.53 (s, 3H, triazole-*H*), 7.43-7.41 (m, 6H), 7.32-7.27 (m, 3H), 6.63 (d, *J* = 8.8 Hz, 6H, C*H*=), 6.59-6.58 (m, 3H, C*H*=), 6.54-6.50 (m, 6H, C*H*=), 4.60 (t, *J* = 6.2 Hz, 6H, CH_2), 4.52 (s, 6H, CH_2), 3.93 (t, *J* = 5.6 Hz, 6H, CH_2), 3.70 (t, *J* = 6.0 Hz, 2H, CH_2), 3.58 (t, *J* = 5.6 Hz, 4H, CH_2), 3.49 (m, 2H, CH_2), 3.41-3.38 (m, 8H, CH_2), 2.87 (s, 9H, NCH_3), 1.37 (s, 27H, *t*-Bu). ^{13}C NMR (100 MHz, $CDCl_3$): δ (ppm) = 172.11, 160.05, 156.92, 149.92, 145.66 (C_q); 137.99, 129.98, 123.64, 123.53 (CH_{Ar}); 115.87, 115.72 (C_q); 113.95, 112.12, 105.93, 102.57 (CH=); 71.36, 71.05, 70.51, 69.29, 64.96 (CH_2); 58.22 (C_q); 52.71, 47.56 (CH_2); 45.37 (C_q), 43.25 (CH_2), 38.82 (NMe), 36.79 (C_q),

[7] R. Ribeiro-Viana, M. Sáchez-Navarro, J. Luczkowiak, J. R. Koeppe, R. Delgado, J. Rojo, B. G. Davis, *Nat. Commun.,* **2012**, 3, 1-9.

28.28 (CH_3, *t*-Bu). HRMS (ESI-HRMS): *m/z* $[M+H]^+$ calcd. for $[C_{87}H_{98}N_{18}O_8Cl]^+$: 1557.7498, 1558.7532; found 1557.7498, 1558.7529.

Synthesis of compound (*EEE*) 3DCM

To a solution of compound **4** (136 mg, 87 µmol) in DMF (5 mL), NaI (0.1 equiv.) and NaN_3 (8 equiv.) were added. The reaction mixture was heated up to 80°C and stirred for 18 hours. After completion of the reaction, the mixture was concentrated and the residue was diluted in EtOAc (20 mL), washed successively with waterand brine, dried over $MgSO_4$, filtered, concentrated and purified by CombiFlash® chromatography with dichloromethane and ethanol (gradient, 100:0 to 90:10, v/v) to yield (*EEE*)-**3DCM** as a red solid (64 mg, 47%).

^{1}H NMR (400 MHz, $CDCl_3$): δ (ppm) = 7.54 (s, 3H, triazole-*H*), 7.41 (d, *J* = 8.7 Hz, 6H, CH_{Ar}), 7.32-7.27 (m, 3H), 6.64 (d, *J* = 9.2 Hz, 6H, C*H*=), 6.58-6.57 (m, 3H, C*H*=), 6.53-6.49 (m, 6H, C*H*=), 4.60 (t, *J* = 6.0 Hz, 6H, CH_2), 4.52 (s, 6H, CH_2), 3.93 (t, *J* = 6.0Hz, 6H, CH_2), 3.64-3.61 (m, 2H, CH_2), 3.58-3.56 (m, 2H, CH_2), 3.52-3.49 (m, 2H, CH_2), 3.42-3.39 (m, 8H, CH_2), 3.33 (t, *J* = 5.2 Hz, 2H, CH_2), 2.87 (s, 9H, NCH_3), 1.37 (s, 27H, *t*-Bu). ^{13}C NMR (100 MHz, $CDCl_3$): δ = 172.07, 160.04, 156.88, 149.90, 145.61 (C_q); 138.01, 129.95, 123.60, 123.42 (CH=); 115.84, 115.72 (C_q); 113.88, 112.07, 105.85, 102.48 (CH=); 71.05, 70.55, 70.08, 69.91, 69.25, 64.91, 58.02 (C_q); 52.65, 50.82, 47.51, 45.33 (C_q); 38.76 (NMe), 36.73 (C_q), 29.79 (CH_2), 28.22 (CH_3, *t*-Bu) ppm. HRMS (ESI-HRMS): *m/z* $[M+H]^+$ calcd. for $[C_{87}H_{98}N_{21}O_8]^+$: 1564.7902, 1565.7936; found 1564.7915.

3. Molecular Dynamics (MD) Simulations

Optimized geometries of (*EZ*)-2DCM and (*EE*)-2DCM

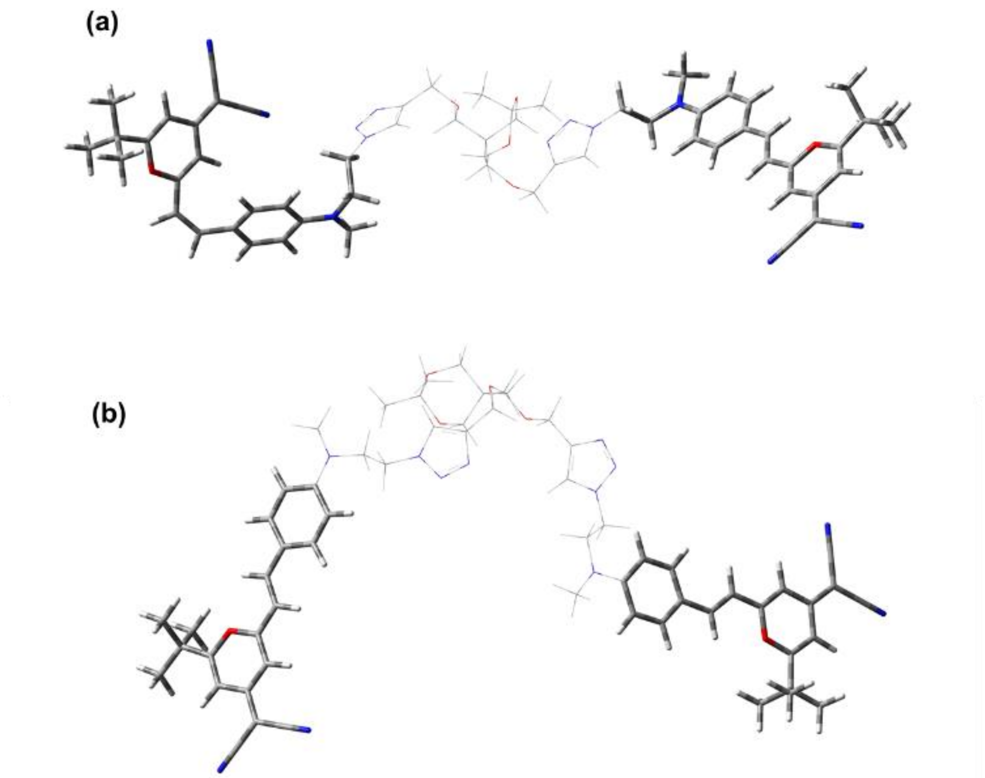

Figure S1. ONIOM optimized initial geometries of (a) (*EZ*)-**2DCM** and (b) (*EE*)-**2DCM** at the {PBE0/6-311G+(d,p):PM6} IEFPCM:THF level. The molecular fragments represented in the tube mode (resp. wire mode) were optimized at the DFT level (resp. PM6 level).

Solvent cubic box with (*EZ*)-2DCM

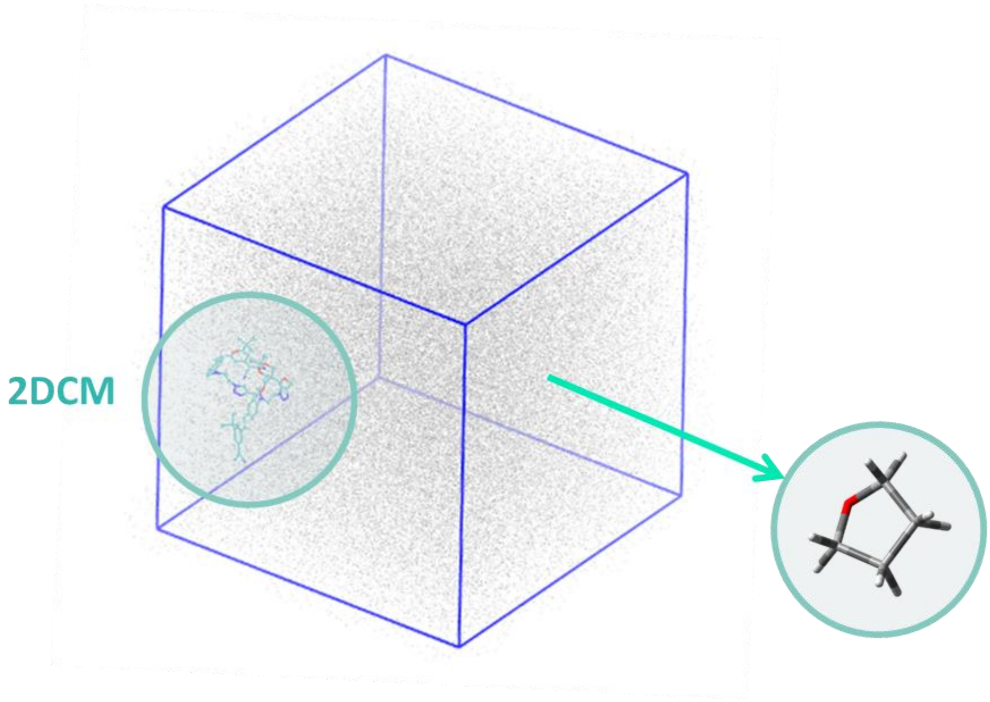

Figure S2. Packmol encapsulated (*EZ*)-**2DCM** with 1500 THF molecules in a cubic box.

Explicit THF molecules were encapsulated with unknown density together with the 2DCMs. The cubic box after the energy minimization was still not similar to the volume observed in the equilibrium state. The cubic box starts simultaneously to compress with the NPT ensemble to the THF density with the constant tau-p = 0.2 ps and 1 bar reference pressure. Finally, we obtain a cubic box with proper density around 0.889 kg L^{-1} at 298K.

To describe the electrostatic interactions, periodic boundary conditions (PBC) were imposed, a Verlet cut-off of 12 Å was considered and the Particle Mesh Ewald (PME) coulomb type was used. For each box, NPT ensembles were run for more than 5 ns with the time step of 2 fs. The production runs are taken after the NPT equilibriums during a total time of 200 ns.

MD data analysis for (*EZ*)-2DCM and (*EE*)-2DCM

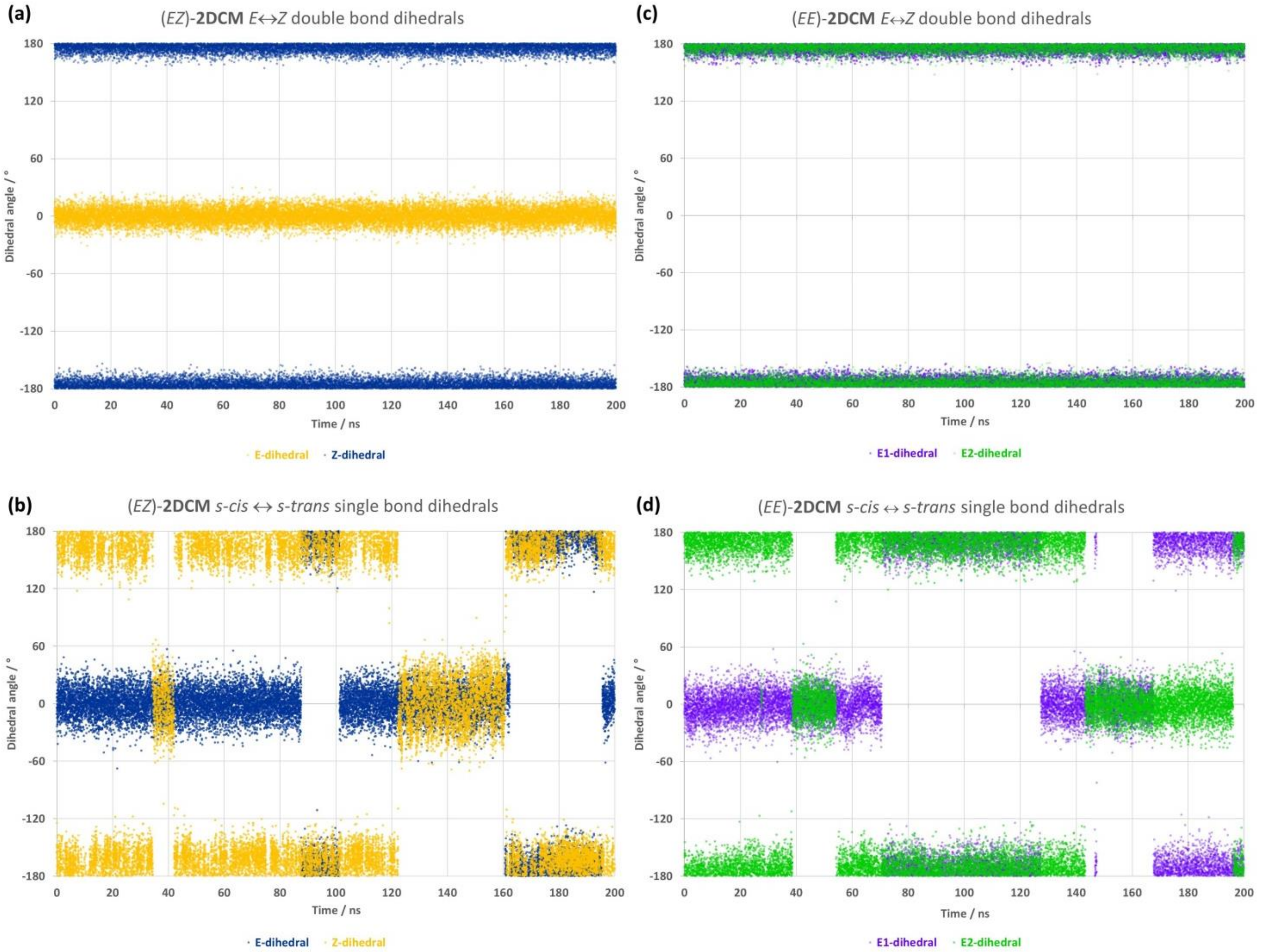

Figure S3. (a, c) MD trajectories of dihedral angles of central C=C double bond controlling the *E*↔*Z* photoisomerization of each DCM unit and (b, d) MD trajectories of dihedral angles of the C-C single bond controlling the *s-cis*↔*s-trans* interconversion of each DCM unit, for (a, b) (*EZ*)-**2DCM** and (c, d) (*EE*)-**2DCM**.

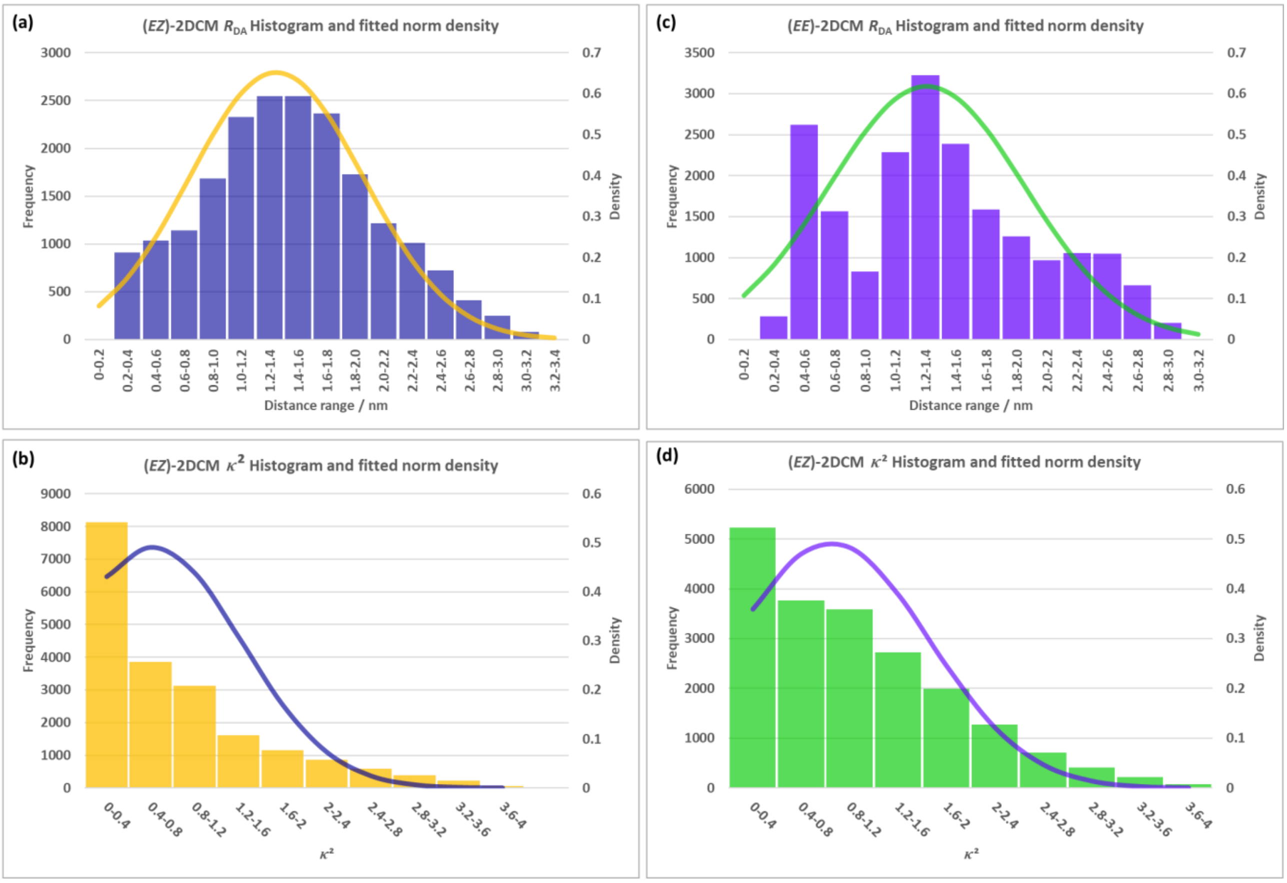

Figure S4. (a) 200 ns (*EZ*)-**2DCM** R_{DA} histogram and fitted norm density. (b) 200 ns (*EZ*)-**2DCM** κ^2 histogram and fitted norm density. (c) 200 ns (*EE*)-**2DCM** R_{DA} histogram and fitted norm density. (d) 200 ns (*EE*)-**2DCM** κ^2 histogram and fitted norm density.

MD trajectories and related data analyses

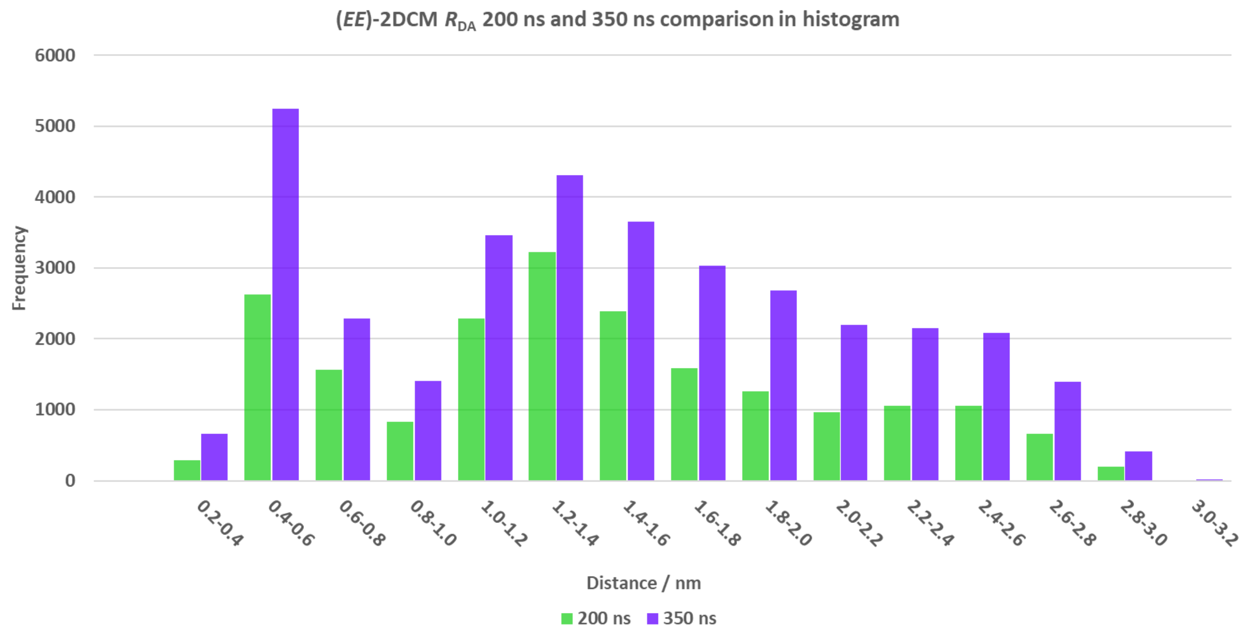

Figure S5. Comparison of R_{DA} histograms of (*EE*)-**2DCM** recorded from 200 ns and 350 ns trajectories, respectively.

Table S1. Time-duration of MD simulations and corresponding average parameters.

**Configuration**	**Time duration**	**Avg. R_{DA}**	**Avg. κ^2**	**Avg. E_{FRET}**
**(*EE*)-2DCM**	200 ns	1.407 nm	1.043	87.8%
**(*EE*)-2DCM**	350 ns	1.446 nm	1.019	85.7%
**(*EZ*)-2DCM**	100 ns	1.476 nm	0.740	61.4%
**(*EZ*)-2DCM**	200 ns	1.446 nm	0.814	63.4%
**(*EZ*)-2DCM**	300 ns	1.451 nm	0.820	64.0%
**(*EZ*)-2DCM**	450 ns	1.404 nm	0.761	65.6%
**(*EZ*)-2DCM**	600 ns	1.448 nm	0.795	64.6%
**(*EZ*)-2DCM**	750 ns	1.437 nm	0.786	64.7%
**(*EZ*)-2DCM**	900 ns	1.392 nm	0.775	66.5%

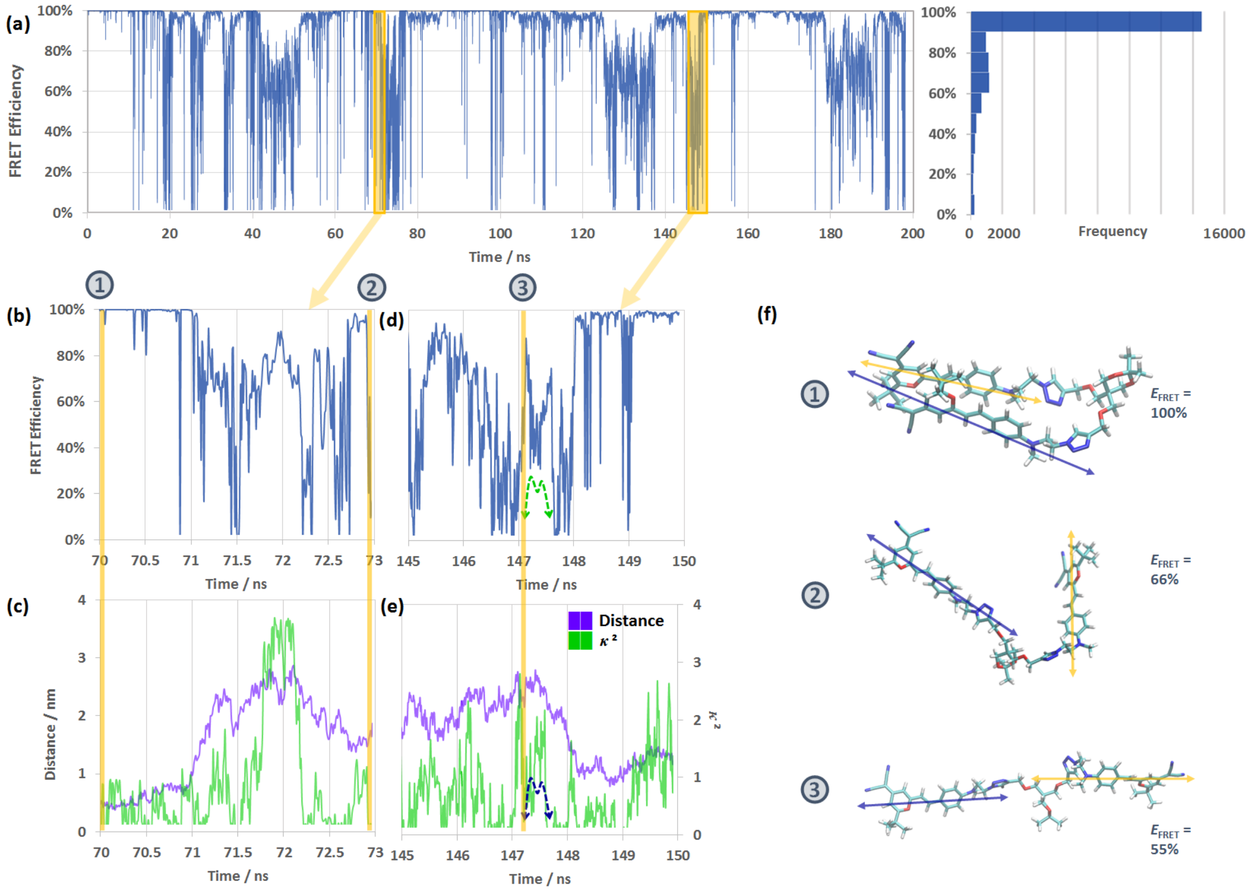

Figure S6. (a) MD trajectory of (*EE*)-**2DCM** homo-FRET efficiency E_{FRET} (t) during 200 ns, and corresponding histogram of E_{FRET} distribution. (b, d) Enlarged areas of the E_{FRET} (t) trajectory in the time intervals 70-73 ns and 145-150 ns. (c, e) Enlarged areas of the R_{DA}(t) (violet) and κ^2(t) (green) trajectories corresponding to (b, d), respectively. (f) Three selected typical geometries of (*EE*)-**2DCM**, indicated by yellow vertical bars in (b-e), with violet and yellow arrows representing the transition dipole moments of the donor (*E*)-DCM and the acceptor (*E*)-DCM units, respectively. Their corresponding FRET efficiencies are marked in the insets.

Free-energy landscape (FEL) based on principal components analysis (PCA)

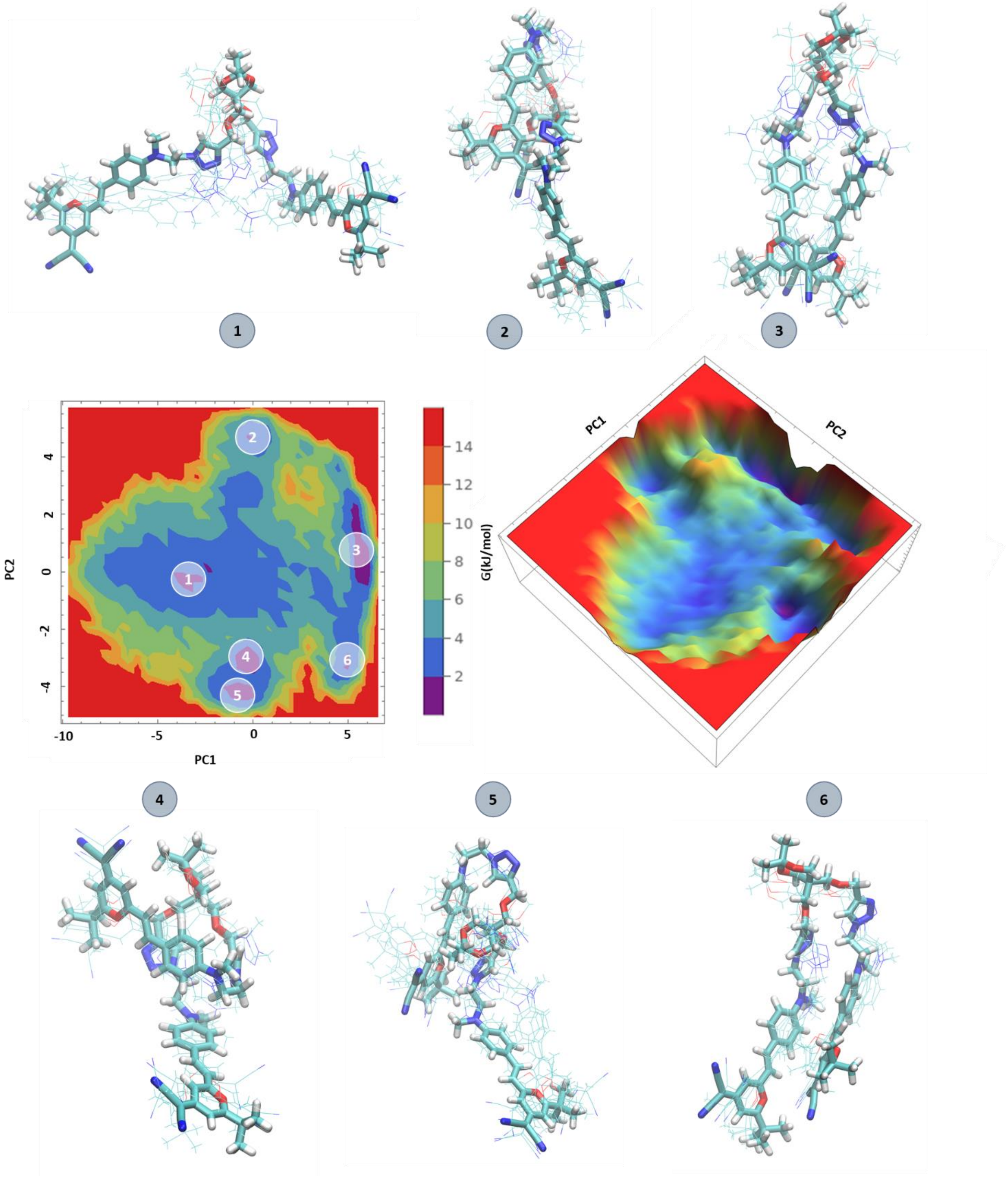

Figure S7. Free-energy landscape (FEL) based on principal components analysis (PCA) of a 200 ns trajectory of (*EE*)-**2DCM**. The represented geometries correspond to each energy valley.

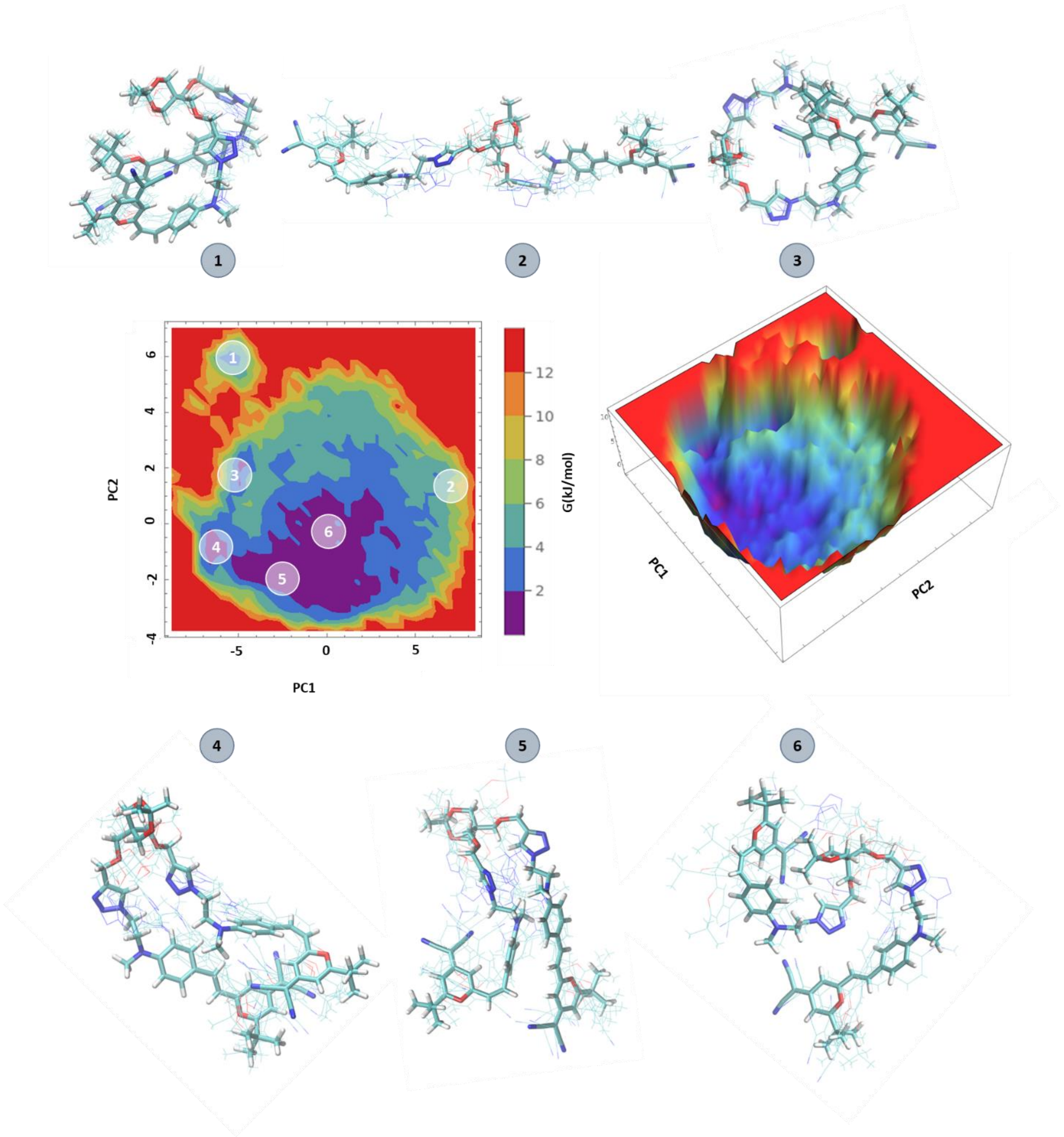

Figure S8. Free-energy landscape (FEL) based on principal components analysis (PCA) of a 200 ns trajectory of (*EZ*)-**2DCM**. The represented geometries correspond to each energy valley.

Implied timescales for the Markov model of (*EZ*)-2DCM and (b) (*EE*)-2DCM

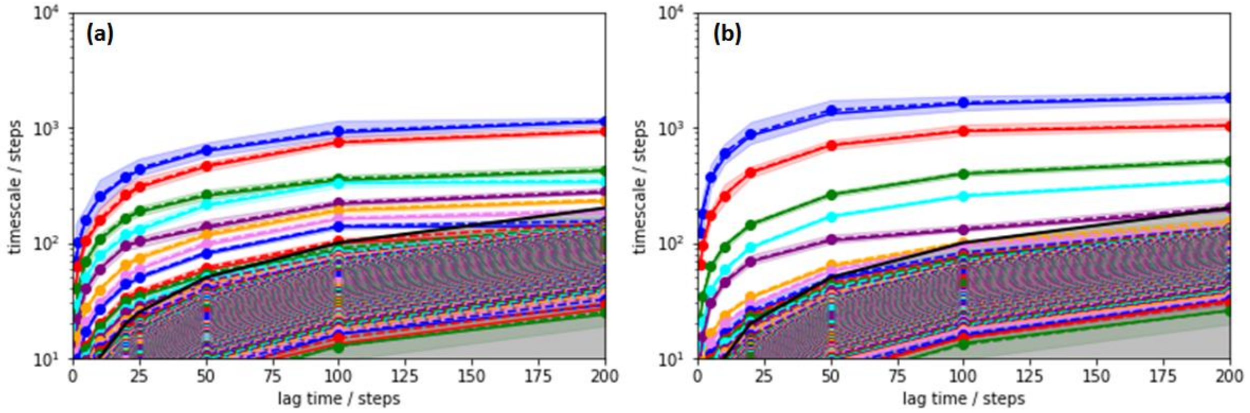

Figure S9. Implied timescales for the Markov model of (a) (*EZ*)-**2DCM** and (b) (*EE*)-**2DCM**. The error bars are 95% confidence intervals estimated using the reversible transition matrix sampling algorithm. The unit of steps has the reduction formula: 100 steps = 1 ns.

Chapman-Kolmogorov test curves of (*EZ*)-2DCM and (b) (*EE*)-2DCM

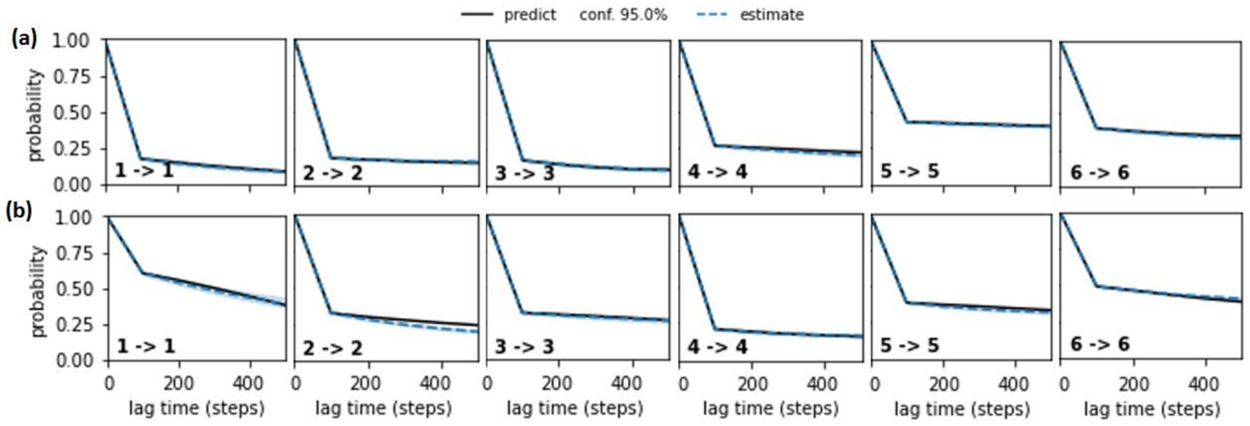

Figure S10. The Chapman-Kolmogorov test curves obtained by 6 macrostates of (a) (*EZ*)-**2DCM** and (b) (*EE*)-**2DCM**. Where "$i - i$" represents the coincidence degree between the predicted proability of the MSM and the simulation probability in i^{th} macrostate. The unit of steps has the reduction formula: 100 steps = 1 ns.

4. NMR spectra

NMR spectra of compound 2 in $CDCl_3$

2,2-Dimethyl-5,5-bis((prop-2-yn-1-yloxy)methyl)-1,3-dioxane (**2**)

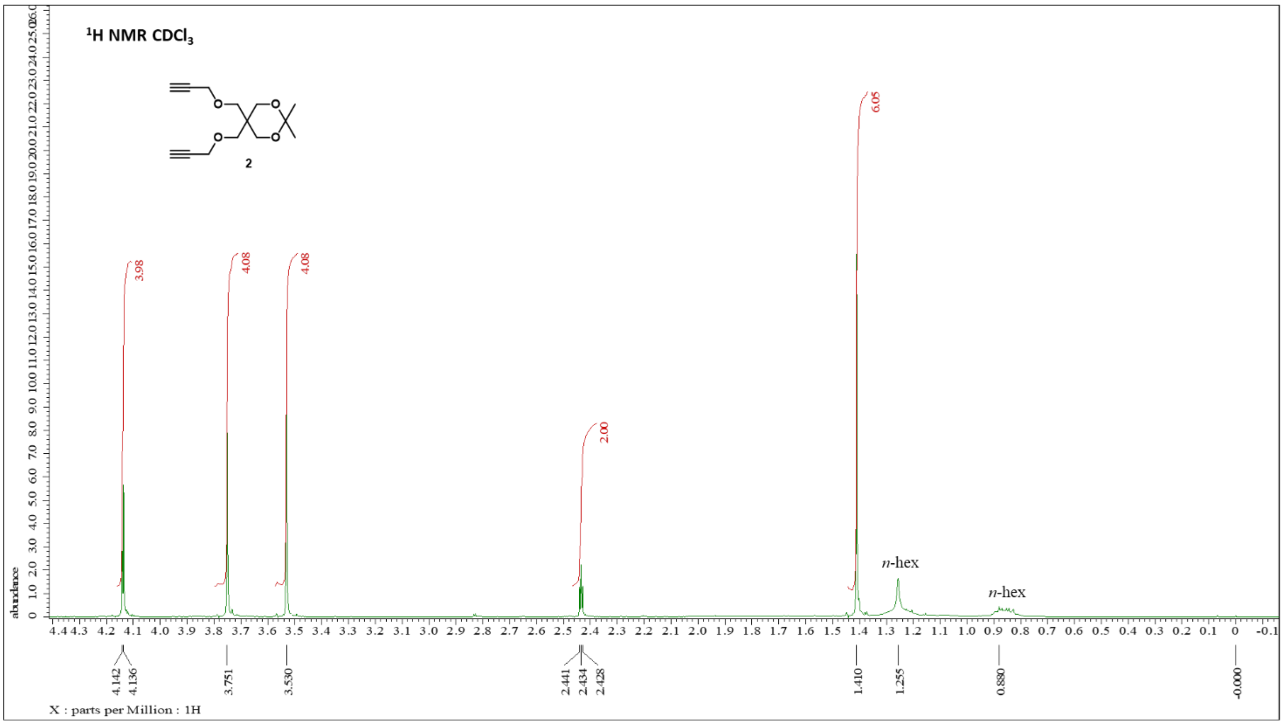

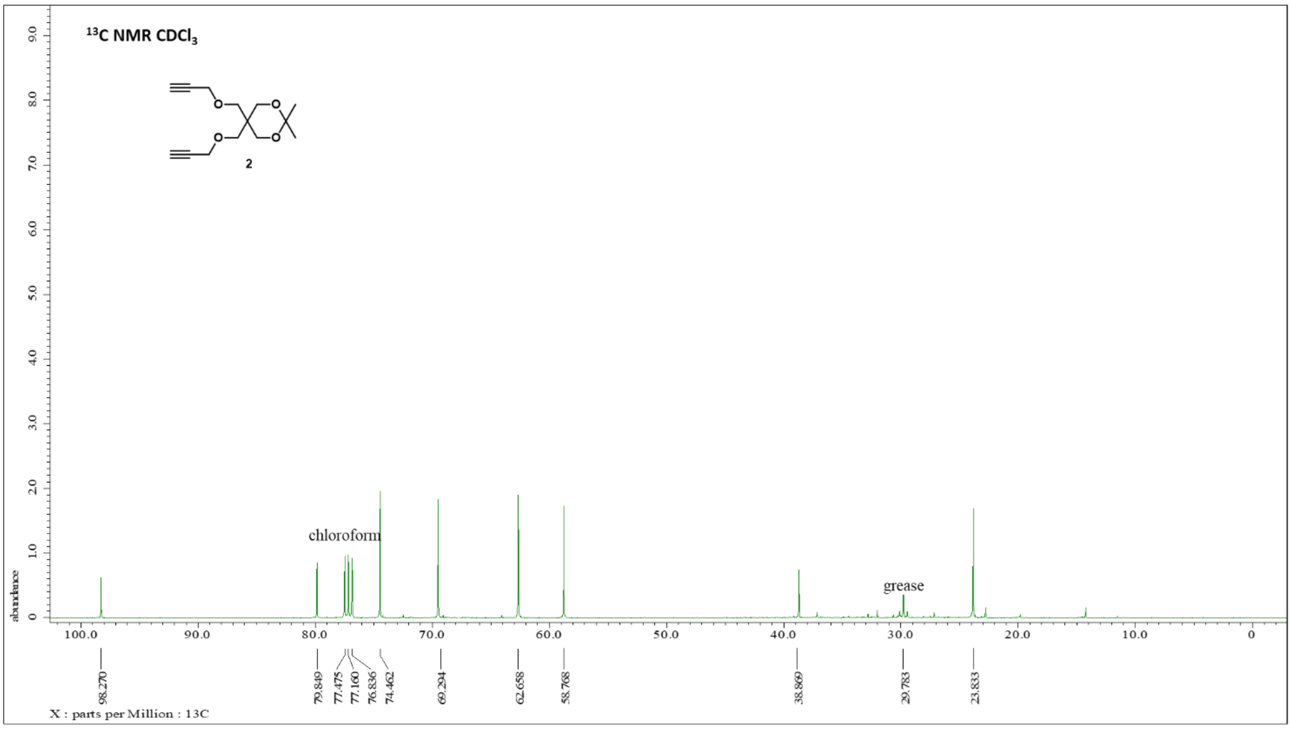

Figure S11. ^{1}H (top) and ^{13}C (bottom) NMR spectra of compound **2** recorded in $CDCl_3$ respectively at 400 MHz and 100 MHz.

NMR spectra of compound (*EE*)-2DCM in $CDCl_3$

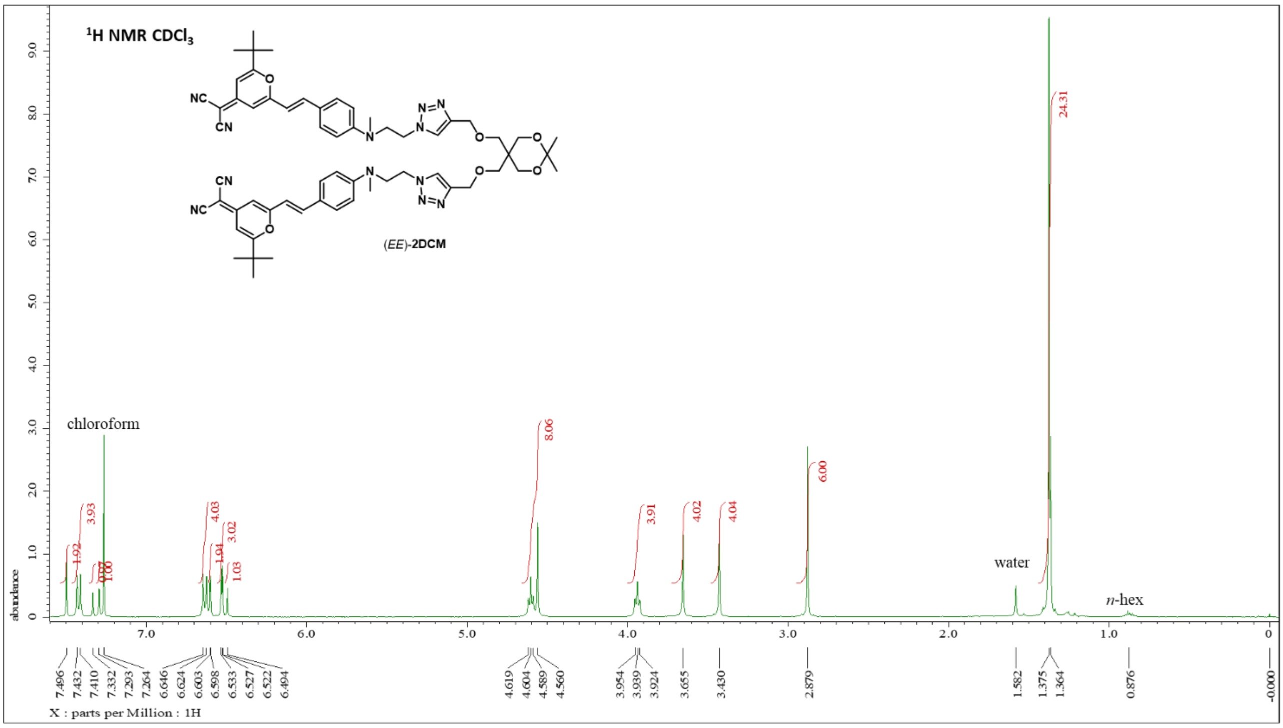

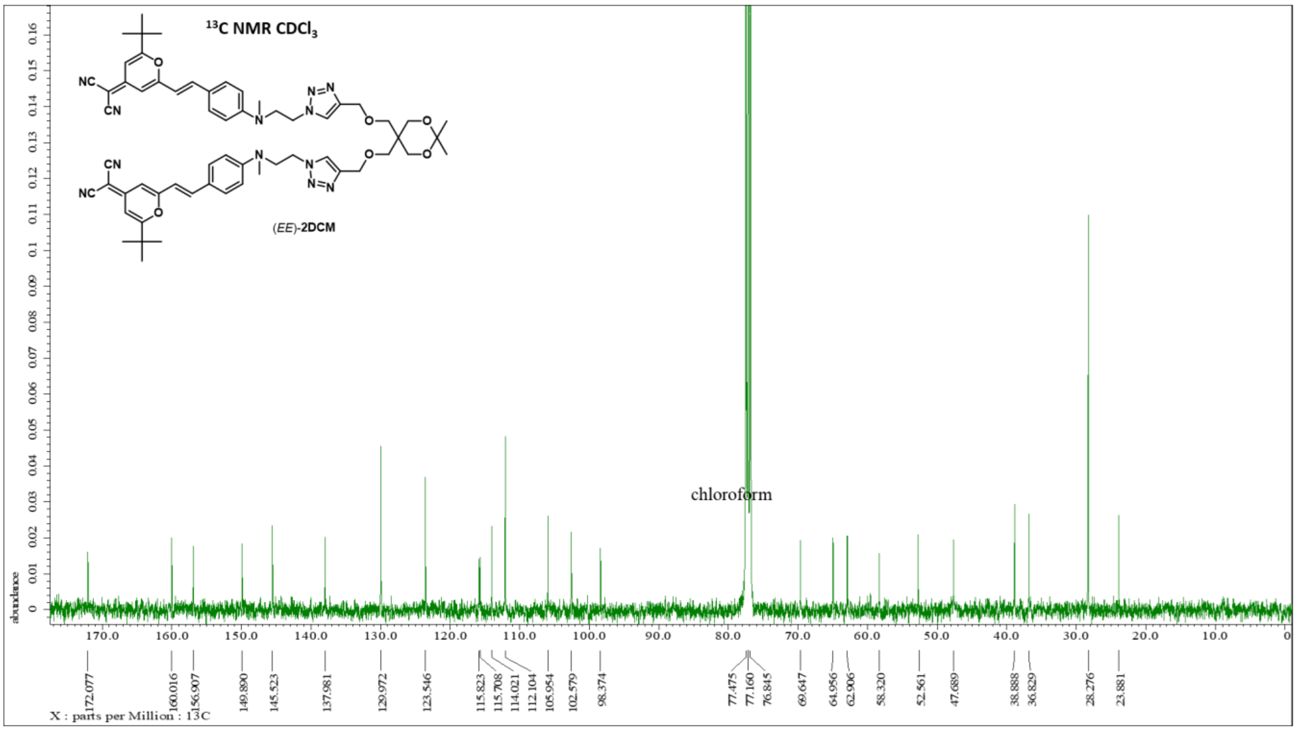

Figure S12. ^{1}H (top) and ^{13}C (bottom) NMR spectra of compound (*EE*)-**2DCM** recorded in $CDCl_3$ respectively at 400 MHz and 100 MHz.

NMR spectra of compound 4 in $CDCl_3$

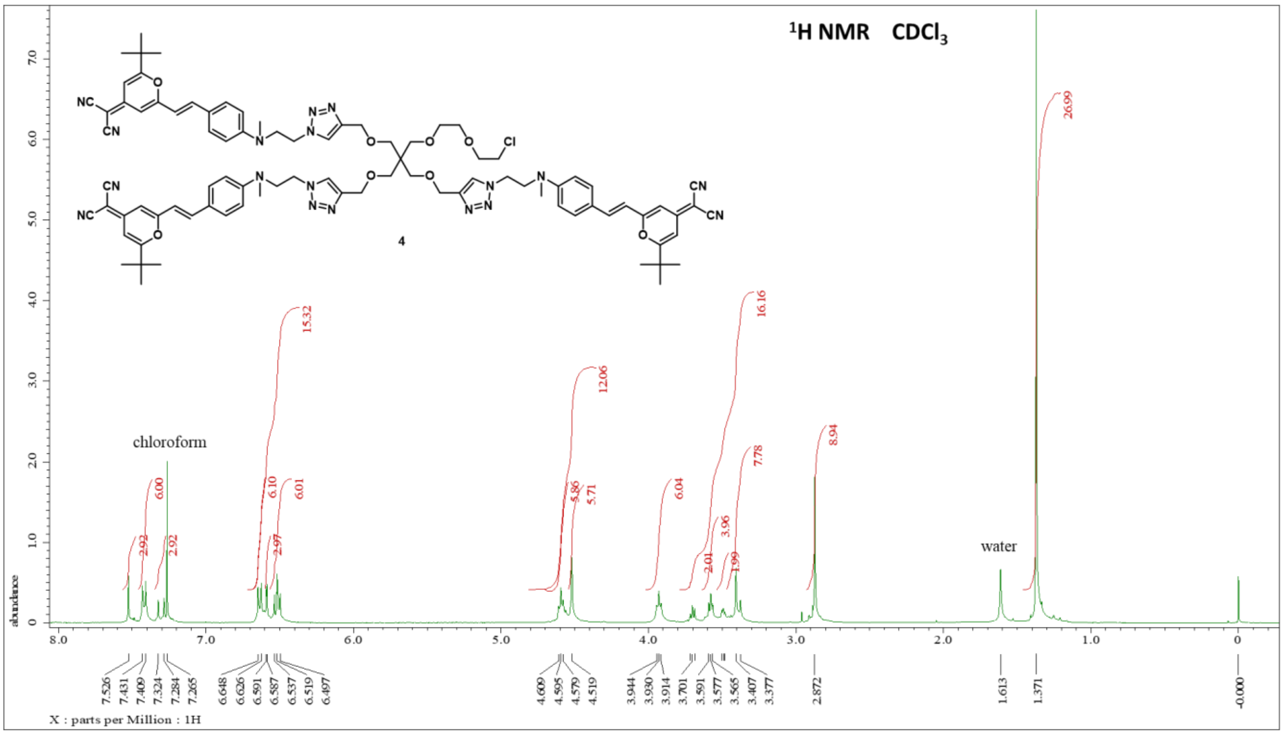

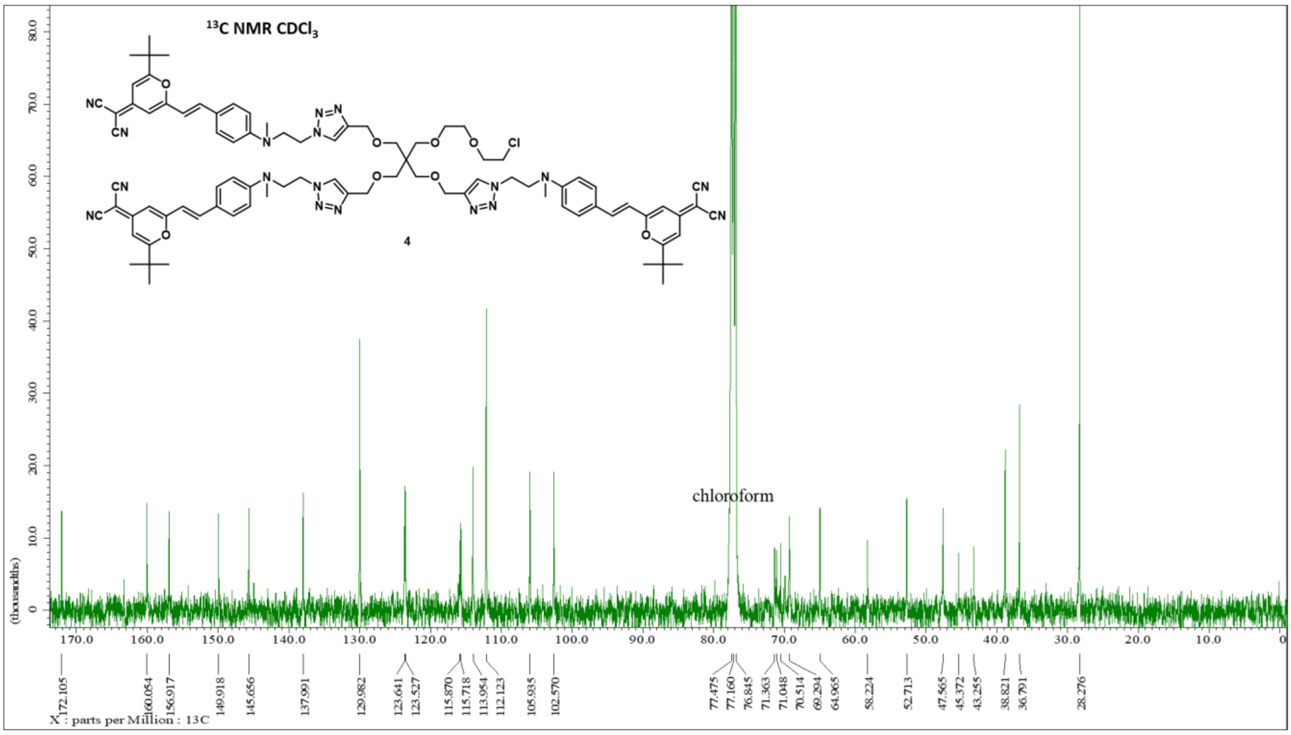

Figure S13. ^{1}H (top) and ^{13}C (bottom) NMR spectra of compound **4** recorded in $CDCl_3$ respectively at 400 MHz and 100 MHz.

NMR spectra of compound (*EEE*)-3DCM in $CDCl_3$

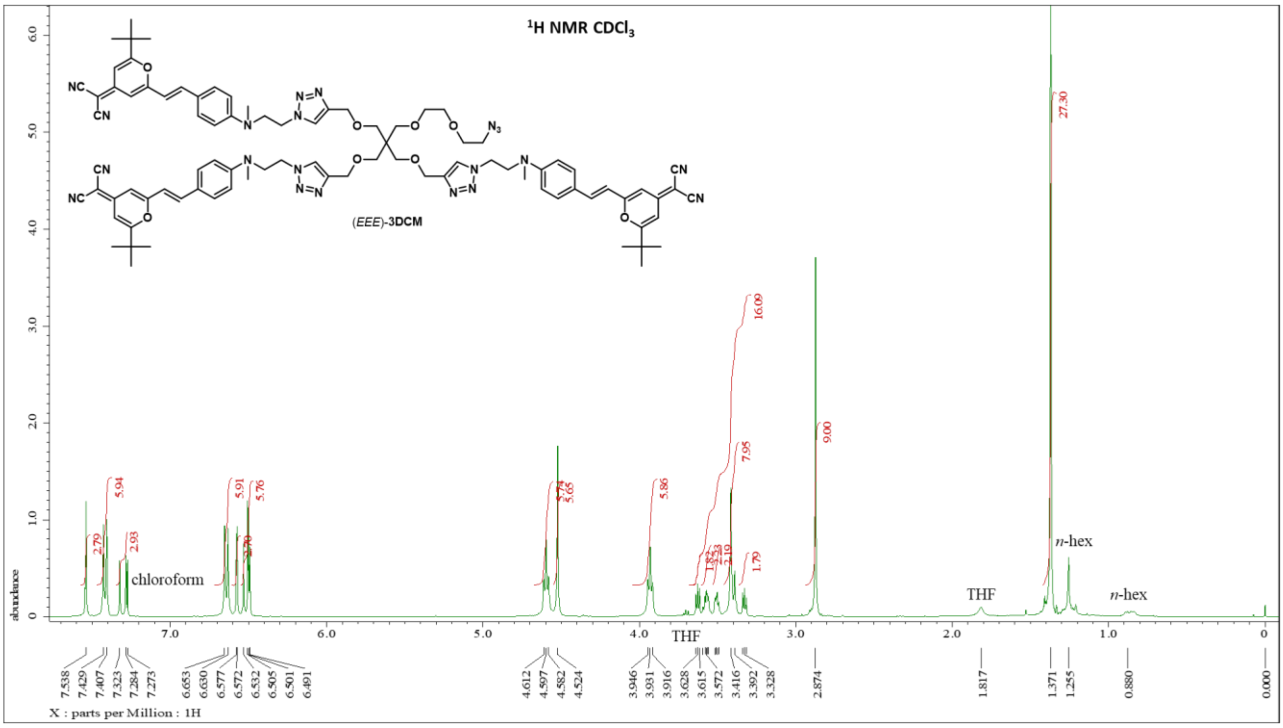

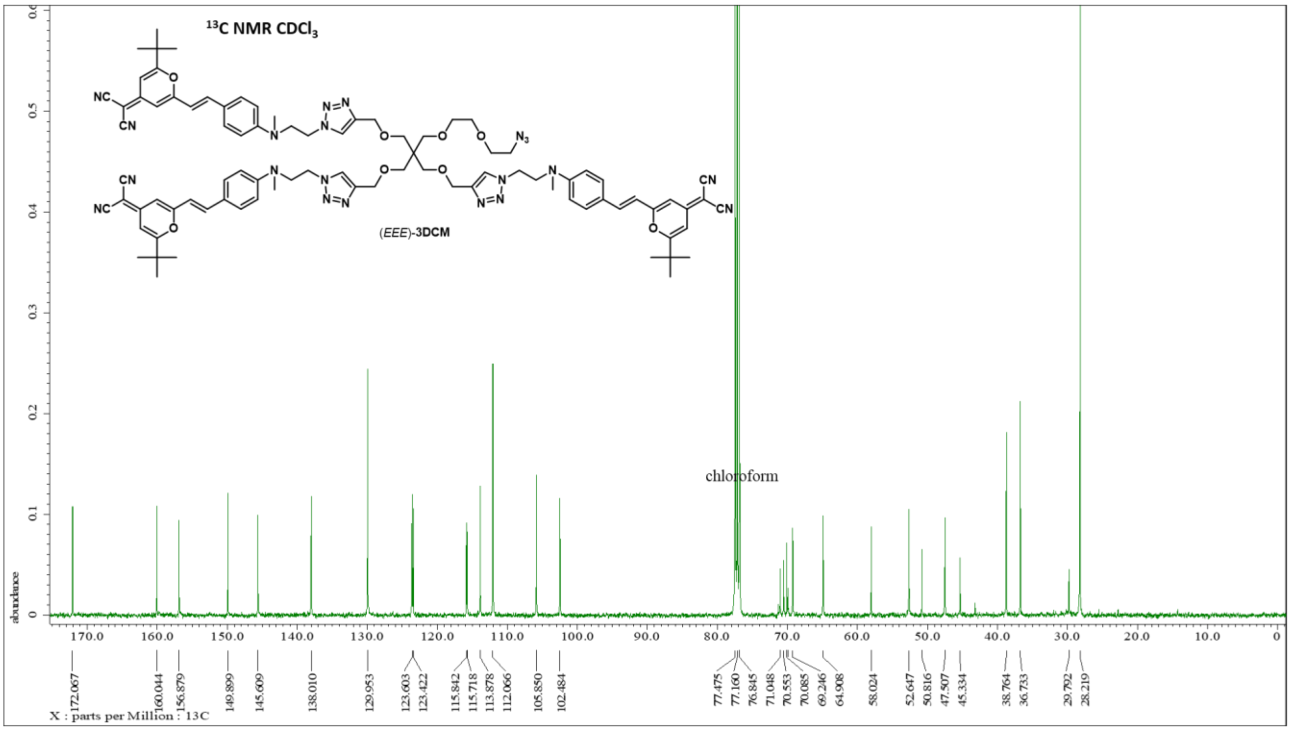

Figure S14. ^{1}H (top) and ^{13}C (bottom) NMR spectra of compound (*EEE*)-**3DCM** recorded in $CDCl_3$ respectively at 400 MHz and 100 MHz.
